# Micro-Surface and -Interfacial Tensions Measured Using the Micropipette Technique: Applications in Ultrasound-Microbubbles, Oil-Recovery, Lung-Surfactants, Nanoprecipitation, and Microfluidics

**DOI:** 10.3390/mi10020105

**Published:** 2019-02-01

**Authors:** David Needham, Koji Kinoshita, Anders Utoft

**Affiliations:** 1 Institute for Molecular Medicine, University of Southern Denmark, Odense 5230, Denmark; koji@health.sdu.dk (K.K.); aum@health.sdu.dk (A.U.); 2 Department of Mechanical Engineering and Material Science, Duke University, Durham, NC 27708, USA; 3 School of Pharmacy, University of Nottingham, Nottingham, NG7 2RD, UK

**Keywords:** micropipette-technique, air-water surface, oil-water interface, soluble surfactant, insoluble lipids, “black lipid films”, “droplet-interface-bilayers”, equilibrium, dynamic, adsorption, gas-microbubbles, oil-microdroplets, lung-surfactants, nanoprecipitation, microfluidics

## Abstract

This review presents a series of measurements of the surface and interfacial tensions we have been able to make using the micropipette technique. These include: equilibrium tensions at the air-water surface and oil-water interface, as well as equilibrium and dynamic adsorption of water-soluble surfactants and water-insoluble and lipids. At its essence, the micropipette technique is one of capillary-action, glass-wetting, and applied pressure. A micropipette, as a parallel or tapered shaft, is mounted horizontally in a microchamber and viewed in an inverted microscope. When filled with air or oil, and inserted into an aqueous-filled chamber, the position of the surface or interface meniscus is controlled by applied micropipette pressure. The position and hence radius of curvature of the meniscus can be moved in a controlled fashion from dimensions associated with the capillary tip (~5–10 μm), to back down the micropipette that can taper out to 450 μm. All measurements are therefore actually made at the microscale. Following the Young–Laplace equation and geometry of the capillary, the surface or interfacial tension value is simply obtained from the radius of the meniscus in the tapered pipette and the applied pressure to keep it there. Motivated by Franklin’s early experiments that demonstrated molecularity and monolayer formation, we also give a brief potted-historical perspective that includes fundamental surfactancy driven by margarine, the first use of a micropipette to circuitously measure bilayer membrane tensions and free energies of formation, and its basis for revolutionising the study and applications of membrane ion-channels in Droplet Interface Bilayers. Finally, we give five examples of where our measurements have had an impact on applications in micro-surfaces and microfluidics, including gas microbubbles for ultrasound contrast; interfacial tensions for micro-oil droplets in oil recovery; surface tensions and tensions-in-the surface for natural and synthetic lung surfactants; interfacial tension in nanoprecipitation; and micro-surface tensions in microfluidics.

## Graphical Abstract


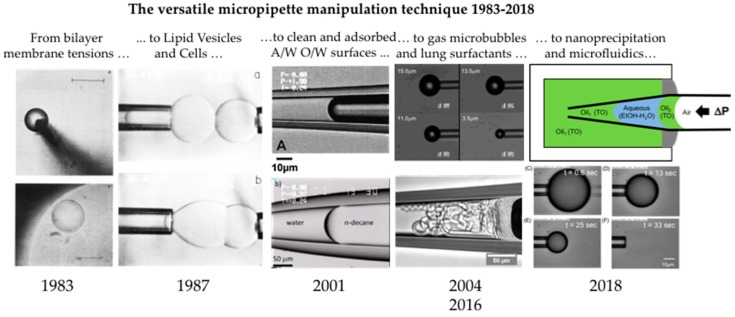


## 1. Introduction

In this contribution to the Special Issue "Microscale Surface Tension and Its Applications," we give an up-to-date review from 1983 [1] to the present day, of our wide range of micropipette-techniques utilised for measurements of surface and interfacial tensions, droplet dissolution, and molecular adsorption in air-water, oil-water, water-oil systems. While there are many other techniques and methodologies associated with microsurfaces including microfluidic tensiometry, capillarity of gas bubbles, and other micro techniques involving “Laplace sensors” [2,3,4,5] we take the liberty to limit the scope of this review to our own studies. Micropipettes are glass capillaries that are custom cut to have tip diameters ~5 μm and can taper out to 450 μm, and so all measurements are actually made on surface and interfacial menisci, gas microbubbles or liquid microdroplets at the microscale. As motivated in the special issue, surface tension and capillary effects enable many of the applications in micro- and nano-systems. So here, by utilising a micropipette technique, we provide direct measures of surface and interfacial tensions at the same scales as microfluidic, lab-on-chip, and other devices. The goal is to provide the readership with a comprehensive review of many of the surface and interfacial tension measurements we have been able to make using the micropipette technique, including equilibrium measurements of the clean air-water surface and oil-water interface [6], as well as equilibrium and dynamic adsorption of water-soluble surfactants [7,8] and water-insoluble lipids [9,10] that required the development of a new technique, the Micropipette Interfacial Area-Expansion Method (MIAM) [7]. We also give examples of where our measurements and those of others [11] have had a direct impact on at least five applications. These selected applications include: gas microbubbles for ultrasound contrast [12,13,14]; interfacial tensions of micro-oil droplets for oil recovery [11]; surface tensions and tensions-in-the surface of natural and synthetic lung surfactants [10]; interfacial tension in nanoprecipitation [15,16,17]; and micro-surface tensions in microfluidics [18].

We start though by presenting a few short stories behind some aspects of surfactancy we find interesting and/or have made contributions to. This historical-perspective briefly describes certain collaborations, personal contacts, and friendships that often underlie, or have even enabled, surfactancy R&D. It takes us on a potted personalised journey that includes: Franklin and his “cruet of oil”; the importance and role of Unilever scientists in generating much of the fundamental studies at the time; an academic-industrial friendship that came out of those studies; an early micropipette technique for studying fundamentals of “Black Lipid Films” (BLMs) and their interfacial tension, and how this system helped to generate a new, and currently very active, field of “Droplet Interface Bilayers” (DIBs).

### 1.1. Franklin and Friends at the Royal Society

As is well known, when an oil droplet is introduced at the air-water surface, an oil film can spontaneously spread producing eventually a monomolecular film. Franklin communicated his famous experiment to his friend William Brownrigg, and it was read and published in the Royal Society in 1774 [19]. While Clapham Common may have been one site for “smoothing the waves,” their friendship extended to Franklin and John Pringle visiting Brownrigg at his home in Ormathwaite in the English Lake District. As recounted by Mertons [20], “The three of them went to nearby Derwent Water where Franklin demonstrated the wave-stilling effect of a little oil he kept in the hollow upper joint of his bamboo cane.”

Having observed that the wakes of two ships were remarkably smooth, while all the others were ruffled by the wind, Franklin asked the captain, who told him that the cooks on those two ships had probably just emptied their greasy water. As recounted by Franklin in his letter to the Royal Society [19,20].

*“At length being at Clapham where there is, on the common, a large pond, which I observed to be one day very rough with the wind, I fetched out a cruet of oil, and dropt a little of it on the water. I law it spread itself with surprizing swiftness upon the surface; but the effect of smoothing the waves was not produced; for I had applied it first on the leeward side of the pond, where the waves were largest and the wind drove my oil back upon the shore. I then went to the windward side, where they began to form; and there the oil, though not more than a tea spoonful, produced an instant calm over a space several yards square, which spread amazingly, and extended itself gradually till it reached the lee side, making all that quarter of the pond, perhaps half an acre, as smooth as a looking-glass.”*

This spreading of an oil film is one of the characteristics of amphiphilic molecules having hydrophilic and hydrophobic parts. As Tanford mentioned in his book in 1980 [21], if the oil molecule has only pure hydrocarbon chains, the film-spreading phenomena will not happen, i.e., the oil stays at the surface as a droplet; but, if it is an amphiphile, a monolayer of just one molecule thick can be obtained, and this monolayer reduces the clean surface or interfacial tension. Hardy, a British biologist and food scientist [22], in his 1912 paper to the Royal Society, entitled “The tension of composite fluid surfaces and the mechanical stability of films of fluid” [23] found that without a polar group (hydrophilic part) in the molecule, there indeed was no driving force for surface adsorption and so no surface tension reduction. Since then, more than one century has passed. Currently, liquid-liquid and liquid-gas interfacial phenomena with various monolayer-forming amphiphilic compounds have been extensively investigated in order to understand the mechanism of spreading, wetting, and dynamic and equilibrium adsorption that change surface and interfacial tension. These fundamental studies have been applied mainly for product development in drug and food industries, which brings us to our next story.

### 1.2. Surfactant-Colleagues at Unilever and Cambridge

It could be argued that one of the biggest driving influences on the basics of surfactancy was in the development of margarine. In relating this story, we have an opportunity to point out that, as is often forgotten when we now so easily collect papers and references from search engines, talented and hardworking people are behind those studies, and it is their legacy from which we now benefit.

The story starts with the establishment of one of the largest industrial mergers of its time; in 1929, Margarine Unie teamed up with Lever Brothers to create Unilever [24]. Unilever scientists were at the forefront of fundamental surface and interfacial chemistry research that underlies the performance of their, now over-400 products in food and beverages (about 40% of its revenue), cleaning agents, and personal care products. One particular group of researchers requires special mention. Brian Pethica and James Mingins, working at Unilever Research, Port Sunlight Laboratory, Port Sunlight, Wirral, Cheshire L62 4XN, England, were particularly prolific from the late 1950s through the 1980s [25,26,27,28,29,30,31,32,33,34,35] (and actually beyond [36]). They published papers on such topics as “The Properties of Ionized Monolayers“ [25]; “Phase-changes and mosaic formation in single and mixed phospholipid monolayers at the oil-water interface” [28]; “Entropies of Compression of Charged Monolayers at Aqueous Interfaces” [29]; “Phospholipid interactions in monolayers” [31]; and “Intermolecular forces in monolayers at air/water interfaces” [35]. These articles appeared in journals like, Trans Faraday Soc, Journal of Colloid and Interface Science and the edited book, “Monolayers“ [29]. These are just a few of their publications on the most fundamental topics in surface science, carried out in a commercial company initially founded on making margarine. We would encourage interested readers to examine these “industrial” papers. 

Interestingly, the “human factor” of research and development is perhaps no better exemplified than in the friendship between the “academic,” Dennis Haydon FRS in the Physiological Laboratories in Cambridge, studying surfactancy of black-lipid films and anaesthesia in biological membranes, and the “industrialist” Jim Mingins at Unilever. They were good friends and colleagues, often referencing each other’s work, and enjoyed especially hiking, and snow- and ice-climbing together. One can only imagine the discussions of monolayers and bilayers, surfactants and margarine, that might have ensued in the crags of Snowdonia in North Wales or Ben Nevis in Scotland [37]. It is therefore an important point to make and recognise that, not only were fundamentals applied in a range of new products since the early part of the 20th century, the development of these applications necessitated advancement in fundamentals of surface and interfacial chemistry. Fundamentals and applications, “academics and industry” went hand-in-hand in those days.

### 1.3. From BLMs in Cambridge (1983) to DIBs in Oxford (2005) and Beyond

One of us, (Needham) was lucky enough to do a post doc with Dennis Haydon FRS from 1980 to 1983. In one project, we evaluated the interfacial tension of a bilayer membrane against water (made from Glyceryl Mono-Oleate (GMO)) and a series of alkanes and squalene [1]. These bilayer membranes were called “Black Lipid Membranes” (BLM), because their thickness is below the wavelength of light and they are only “visible” microscopically at reflected glancing angles. The goal of the experiment was to evaluate the interfacial tension of a bilayer membrane (*σ*) by measuring the interfacial tension of the stabilising monolayer (*γ*) and the contact angle (*θ*) between a lens trapped in the bilayer, and with it, determine the free energy of formation of the newly discovered solvent-free films in equilibrium with Squalene, as first introduced by Stephen White [38]. In the methods previously developed by Haydon et al., the black lipid membranes were formed in a tiny 1 mm-diameter hole drilled in a Teflon support [39] and GMO-decane solution was introduced into the hole via painting across the hole with a small paint-brush. Normally, for BLMs formed by solutions like GMO-decane, hydrocarbon-lenses were readily trapped in the bilayer during the electrical compression that triggers bilayer formation and the angle was found to be ~2° [39]. The contact angle was simply measured from the ringed-interference pattern of the lens, (or torus) when viewed in incident light [39]. However, for systems where the difference in bilayer to monolayer tension was greater, we expected a much greater contact angle. It was nevertheless thought that the interference fringes could be observed and measured using a high resolution interferometer, in the labs at Port Sunlight. However, with the GMO-Squalene systems, we found that it was not possible to even trap lenses during this process associated with bilayer formation. Attempts failed to form a lens. Repeated failed attempts, one after the other, were followed by a cleaning of the hole in which the GMO solution was introduced in the Teflon support, (i.e., quick “suck-blow” with a pasteur pipette), ready for the next attempt. One night, a surprising (and as it turned out fortuitous) observation was made; when the next bilayer was formed, (without a trapped lens), over the next few minutes, tiny spots of light appeared in the membrane when view in incident light. Remarkably, what had happened was that, the “suck-blow-cleaning” with the pasteur pipette had formed small emulsion droplets, that were driven downwards. 

Then, when a new bilayer was formed, and as the emulsion droplets rose due to their buoyancy-effect, they touched and fused with the new bilayer! Tiny lenses had formed! A new apparatus had to be designed, built, and tested. 

**Figure 1:**
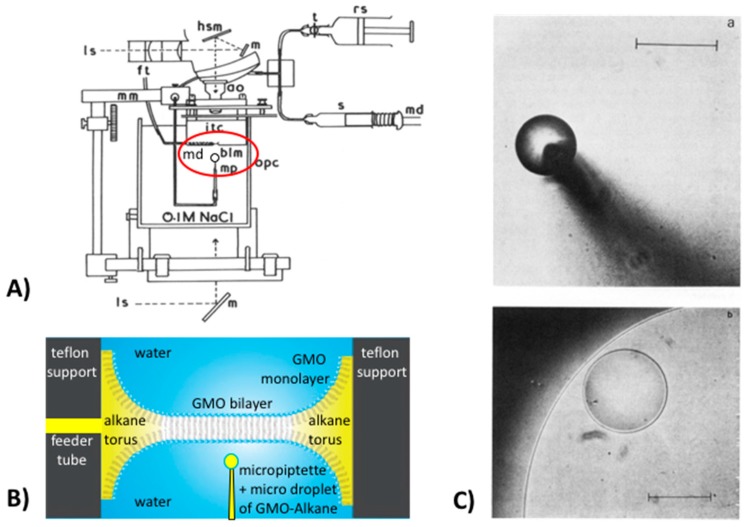
Measurement of high contact angles in black lipid membranes. (A) Diagram of the apparatus showing the micropipette (mp) with microdroplet (md) of Glyceryl Mono-Oleate (GMO)-alkane placed just beneath the black lipid membrane (blm) circled in red. (**B**) Schematic of the BLM formed in a Teflon support using a feeder tube, showing the GMO bilayer in the hole, in equilibrium with the GMO monolayer on the alkane torus, and the positioning of the micropipette with microdroplet ready to be inserted into the bilayer. (**C**) Photographic images of: (a) a droplet of monoolein 8.4 mM in squalene under 0.1 M NaCl on the end of a micropipette of tip external diameter 13.6 µm; (b) the lens that was formed by touching the droplet to the black lipid film formed from the same solution. Bar equals 100 µm. Adapted from [1].

A more controlled technique was therefore developed in which a single, new lens could be formed in the bilayer by introducing it to the bilayer interface with a micropipette using an in-house built contraption [1], as shown in Figure 1A. When the lens was viewed with incident interference optics, the interference fringe-rings were not resolvable, implying that the contact angle was indeed quite large. However, as shown in Figure 1C, the hydrocarbon-filled-torus that supports the bilayer can be viewed in transmitted light, as could the lens. And so, the new method that was developed as a consequence of this “happy accident,” allowed a lens of known volume to be ever-so-gently, introduced from underneath these, notoriously fragile (“2-molecule-thin”) bilayers by the micropipette (Figure 1B). Then, by viewing in transmitted light (Figure 1C), we could visualise (and photograph) the new lens, and use its geometry to calculate its curvature and hence contact angle with the bilayer [1].

Thus, this apparatus and technique [1] allowed us to measure the actual membrane tension, *σ*, given by

*σ* = 2*γ*cos*θ*. (1)

Then, from the area of the film A, we determine the Helmholtz free energy of formation of a black lipid film, Δ*A*, from the relationship,

(2) ΔA=2γA(cosθ−1).

For the solvent-free films in equilibrium with squalene, [38] (or with Triolein [1]), the contact angles were much higher than for the solvent containing decane films (of only 2°). The squalene monolayer tension was measured by the drop-volume method to be 2.4 mN/m, its contact angle was 26.5°, giving a bilayer tension of 2.2 mN/m and a Helmholtz free energy of formation per unit area ΔA* of −511 + 134 μJ/m^2^ which was ~100 times that of the decane film (only −4.5 μJ/m^2^). For GMO bilayers in equilibrium with Triolein, the difference between the tensions of the monolayer (1.8 mN/m) and bilayer (1.0 mN/m) were even larger, showing an even larger contact angle (57°) and concomitantly larger free energy of formation, of −1673 μJ/m^2^. Thus, for these solventless bilayers of only 2.3 nm thickness (essentially twice the length of the GMO hydrocarbon oleate chains), the free energies of formation and hence stability turned out to be very high. Previous explanations for the free energy of formation of membranes made from GMO-decane to -hexadecane measured previously by Requena et al. [40], had focused on simply the thinning energy associated with the Lifshitz theory of van der Waals attraction of water across the membrane. However, the newly-measured values supported the molecular-exclusion, mean field theory predictions of David Gruen, (who was also a post doc of Haydon’s) 3 years earlier working on theory [41,42]. These solventless bilayers then provided artificial, solvent-free model membranes [38] that more closely modelled natural membranes. Thus, in 1981, Needham was already working with, and exploring the use of, a micro-manipulated-micropipette to deliver oil droplets for interfacial tension measurements long-before working with the current more advanced micropipette manipulation set up (see later, Figure 4). In fact, it was this ability to innovate, develop and perform these very delicate experiments that prompted Haydon to suggest and recommend Needham (for his next post doc) to Evan Evans, who had been pioneering the micropipette manipulation techniques for evaluation of red blood cell membranes since the early 1970s. And, as they say, the rest is history. 

**Figure 2:**
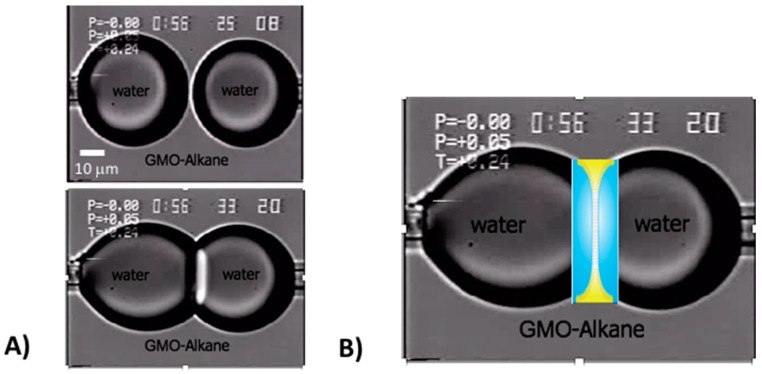
Two opposing water droplets under oil. (**A**) Two water droplets formed (top) in GMO-Alkane solution have a monolayer of GMO at their interfaces. When presented to each other (bottom) using micropipette manipulation they form a GMO-bilayer between them; (**B**) the same image as in (A) (bottom) but now with an overlaid-schematic of the Droplet Bilayer Interface (DIB) showing that a bilayer is formed between them as in the black lipid film.

Finally, it was recognising this new-found bilayer stability that led Needham, in the summer of 2005, to suggest to Hagan Bayley, Professor of Chemical Biology at Oxford University, to flip the BLM-system and use two opposing water droplets under oil and so form a similar bilayer between them [43]. Bayley had been studying single-channel conductance of hemolysin channels in BLMs, but was having trouble stabilising the films. As illustrated in Figure 2, Needham had shown in, as then, unpublished work, that when two water droplets were formed on the ends of two micropipettes in solutions of GMO-alkane and then brought gently into contact, they spread on each other but did not fuse into one droplet. It was clear that a “BLM” had been formed between the two microdroplets (Figure 2A) and was stabilised in this droplet-droplet system as shown in the microscope image and schematic bilayer overlay in Figure 2B.

Upon learning this, Bayley’s post doc, Mathew Holden, rapidly and successfully implemented the idea [44] and this two-droplet system joined by a black lipid film became known as the Droplet Interface Bilayer (DIB). Since then the DIB has been the basis for and invention [45], and multiple new applications involving membrane-stabilised protein channels [43], and the development of a new class of active material based on the ion-transport properties of functional biomolecules [46]. Adding more and more droplets together, Gabriel Villar then created new droplet networks as “Multisomes” [47], that were shown to make tissue-like printed-droplet-networks [48,49] of 35,000 droplets and their stabilising interface bilayers [48]. As Villar concluded, (these networks) “might be interfaced with tissues, used as tissue engineering substrates, or developed as mimics of living tissue” [48]. These systems have also been extended by others into droplet microfluidics for the construction of compartmentalised model membranes [50], and organogels [51]. Here then, this simple BLM, when flipped to be a bilayer between water droplets in oil (Figure 2B), has now spawned over 1000 entries listed on Google Scholar. Once again, this kind of innovation, research, and development exemplifies the power of developing new techniques to allow new measurements—in this case of bilayer tensions, understanding fundamentals of free energies of formation, that are picked up by other talented and driven scientists (perhaps our friends, or friends to be) for subsequent development and new applications. We hope you enjoyed this little potted-history of just some aspects of surfactancy and appreciated the “human factor” at the root of all published research that we perhaps too often take for granted. 

## 2. Basic Micropipette Manipulation Techniques for Surface and Interfacial Tension Measurement

The micropipette manipulation technique is based on the principles of capillary action. As is well-known, capillary action is the tendency of a fluid to be raised in a narrow tube, as the result of the positive adhesion and wetting of the tube by the liquid. (Note: Non-wetting can produce the opposite effect and suppress capillarity, e.g., mercury-air-glass). The classic observation is that, when a narrow glass tube, with a radius of a few hundred microns, is dipped into water, the water rises up the tube to such an extent that its wetting-adhesion around the circumference of the glass opposes the gravitational force on its raised mass. In the micropipette technique, we use similar glass capillary tubes mounted horizontally (hence, no gravity effects) where the capillary action is now precisely controlled by the application of often delicate, applied micropipette pressures (10 s of micro-metres of water) to sometimes quite forceful (10 s of centimetres of water), all viewed under an inverted optical microscope. While initially developed in its current form in the early 1970s and used for studying the micromechanics of red blood cells [52,53,54,55,56,57,58,59,60] white blood cells, [61,62,63,64,65,66,67,68,69,70,71], and lipid vesicles [72,73,74,75,76,77,78,79,80,81,82,83,84], here, we review its adaptation for measurements of surface and interfacial tension at air-water surfaces, oil-water interfaces, and the equilibrium and dynamic adsorption of surfactants and lipids. 

### 2.1. Principles of Capillary Action and the Micropipette

**Figure 3:**
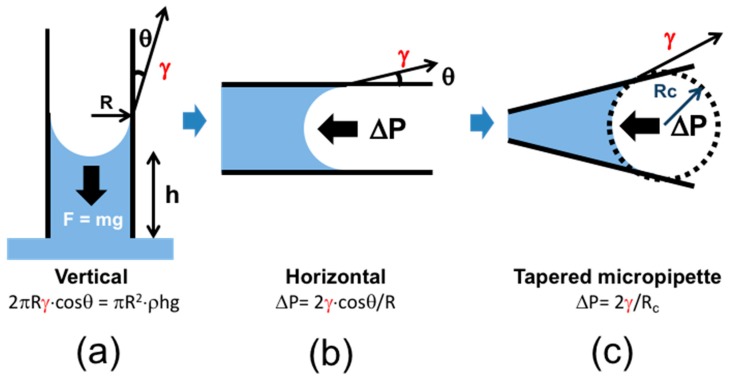
Capillary actions of the air-water surface. (**a**) Capillary-rise of water inside a vertical capillary. Upon dipping the capillary tube into the liquid, meniscus rises against gravity a distance h above the outside bulk surface. At equilibrium, this height reaches a force balance that follows the Young–Laplace equation between gravity and wetting (mg versus 2π*Rγ*cos*θ*). (**b**) In the horizontal capillary immersed in an aqueous-filled microscope chamber, the water comes inside the horizontal capillary until opposed by a counter force such as applying a pressure to the back-end of the pipette to hold it in position or push it back. Again, the force balance follows the Young–Laplace equation of pressure opposed by the surface tension scaled by the reciprocal of the single radius. (**c**) Similarly, for a tapered micropipette where the meniscus position is now opposed requiring a series of increasing applied pressure to move the surface down the taper. It is this tapered pipette that has been the most useful and is the basis for our tension measurements.

At its essence then, the micropipette technique is one of capillary-action, glass-wetting and applied pressure. Classical capillary rise is a well-known physical phenomenon associated with the surface tension forming inside a capillary [85,86,87]. It relies on gravity as the opposing force on the water that wets the glass capillary at its air-water surface meniscus. In general, when one end of a vertical capillary is immersed in a liquid to from the air-liquid surface, the liquid comes into the capillary. Figure 3a shows a schematic image of this capillary action. 

In the vertical set up (Figure 3a), the liquid-rise is due to wetting of the glass (at some wetting contact angle), providing a concave surface. This means that the pressure just below the surface is less than the ambient pressure above it. The height of the meniscus (*h*), from the bulk surface is thus the equilibrium between this reduced pressure scaled by the interfacial tension-acting at the capillary circumference effectively “pulling” the meniscus upwards and gravitational force of the mass of liquid “pulling” downwards. Thus, the interfacial tension force (*F_i_*) is expressed by the equation, *F_i_* = 2π*Rγ*cos*θ_c_*, where *R* is the inner radius of the capillary, and *θ_c_* is the contact angle between the liquid and the capillary surface material. The gravitational force, as a counter balance force, is expressed by, *F_g_* = π*R*^2^*ρh*g, where *ρ* is the density of the liquid, and g is the gravitational constant. Using these equations, the interfacial tension is given by the following relationship [87],

(3) $$\gamma =\frac{R\rho hg}{2\cos\theta_{c}}.$$

From the proportional relations of *R* and *h* in this equation, the capillary rise becomes higher, when the capillary radius becomes smaller. The relation shows that for a given liquid, material of the capillary (e.g., glass), and air-water surface tension, the capillary radius is a key factor in controlling the capillary rise of the meniscus surface. 

Using these principles, micropipette capillary techniques were developed to observe the gas-liquid surface or liquid-liquid interface in an inverted microscope. Micropipettes are mounted horizontally as a parallel shaft (Figure 3b) or tapered shaft (Figure 3c). Now, the position of the surface or interface meniscus is controlled by the applied micropipette pressure, and can be moved in a controlled fashion all the way to dimensions associated with the capillary tip (~5–10 μm) (and even blow out a bubble, see later, Figure 21). Figure 3b shows the capillary in a horizontal position and thus there is no gravitational force acting on the meniscus or liquid in this position. In this case, the liquid comes inside the capillary by capillary action unopposed and would move throughout the whole length of the capillary tube since there is no counter balance against *F_i_*. To resist the liquid flowing into the capillary, a counter balancing force can be initiated inside the micropipette by applying a positive (blowing) pressure, which, when scaled by the surface tension and reciprocal of the radius, is again the Laplace pressure. The relation is thus described with the Young–Laplace equation,

(4) $$\Delta Ρ=\frac{2\gamma \cos\theta_{c}}{R}.$$

The Laplace pressure required to stop the liquid flow is proportional to the reciprocal of the capillary radius, and so, again, smaller diameter capillaries require higher opposing pressures to create smaller radii of curvature. The other important factor is the contact angle *θ_c_* between the liquid and the glass-surface material of the capillary. The contact angle of the air-water surface at a clean glass surface is about 5°. To apply this technique for all liquids including mixed solutions against glass surfaces and other surfaces, the *θ_c_* values have to be measured independently. However, because of the constant diameter of the parallel capillary there is only a single pipette pressure for any given tension, and so statistical averaging is somewhat limited. To allow multiple pressure-radius measurements and so provide self-consistent (same system) values for the surface or interfacial tension, in 2001 a new capillary-action-based technique, the tapered micropipette, was developed and applied to a series of clean surfaces and interfaces as well as soluble- and insoluble-surface active materials [6,9].

Figure 3c shows the schematic image of the tapered micropipette for a surface tension measurement. The tapered capillary is again set in a horizontal position. Following the Young–Laplace equation and geometry of the capillary, the Laplace pressure controlling the liquid flow shows the more simple relation (Young–Laplace equation),

(5) $$\Delta Ρ=\frac{2\gamma }{R_{c}},$$

where, again, *R_c_* is the radius of curvature of the interface inside the capillary. Using the tapered shape for the capillary, the factor of *θ_c_* is cancelled out in the equation, as discussed below in association with Equation (6).

Therefore, the surface or interfacial tension value is obtained by knowing the applied pipette pressure Δ*P*, required to keep the meniscus at a geometry of *R_c_*, and does not require knowledge of the contact angle of the meniscus at the capillary surface.

### 2.2. Micropipette Manipulation Apparatus

**Figure 4:**
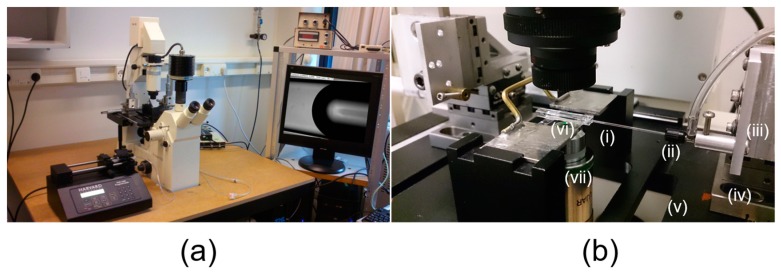
Our Signature Micropipette Manipulation Platform provides an ability to establish a well-defined interface (air-water, oil-water, with or without adsorbed material). (**a**) The system is comprised of: micropipette pressure control from micro-atm to milli-atm; a microchamber for the test solution; temperature control: 5 to 50 °C; manipulators with fine positional control of pipette. Microscope images are recorded digitally for analysis. (**b**) Micropipette (i) is mounted via a chuck (ii) in a custom-built holder (iii), mounted on a stage micrometre (iv) bolted firmly to the microscope platform (v). The image of the micropipette in the microchamber (vi) is viewed via a 40× and 20× objective lens for equilibrium and dynamic surface tension measurement, respectively (vii). With permission from Elsevier [18].

The interfacial tension measurement with the tapered micropipette is achieved by using a bright field microscope system with one or more micropipette micro-manipulators mounted on the microscope stage [7,9]. Other accessary equipment, such as a pressure transducer and camera are also required. Figure 4a shows a photographic image of the micropipette manipulation system with a tapered micropipette mounted in a microscope chamber filled with the test liquid (Figure 4b).

Figure 4a shows the overall microscopy system. The bright-field microscope with Köhler illumination (Zeiss) is used to observe the geometrical shape of the surface or interface inside a micropipette, as for example, the water-air surface displayed on the monitor in Figure 4a. Figure 4b shows an enlarged view around the glass cuvette sample-chamber (2 mm × 3 mm × 10 mm) with a micropipette inserted. Tapered micropipettes (taper angle *θ_p_* ~ 3–4°) are custom-made de novo by using a pipette puller (Shutter instrument, Novato, CA, USA), and cut to the desired tip diameter (~5–10 μm) with a micro-forge (Narishige) [18]. To manipulate the tapered micropipette inside the chamber, the pipette is attached to a Newport 3D mechanical micromanipulator, which is firmly bolted to the microscopy stage. To monitor and record the geometrical shapes at the tip and the interface inside the tapered micropipette, a CCD-camera with 30 frames per second (DAGE-MIT, Michigan, IN, USA) is attached to the system. In-line pressure transducers (Validyne Engineering Corp., Northridge, CA, USA) measure the applied pipette pressure, in the plastic tubing that connects to the micropipette via an “L”-junction chuck. Precise pressure control by a syringe or manometer allows the system to be set to zero flow and hence zero applied pressure. Surfaces, interfaces and formed gas microbubbles or liquid microdroplets are monitored in real time and recorded as digitalised information on a computer by using a home-built LabVIEW program. The digitised image is analysed with ImageJ software provided by National Institute of Health [88].

### 2.3. Gas-Liquid Interfaces

**Figure 5:**
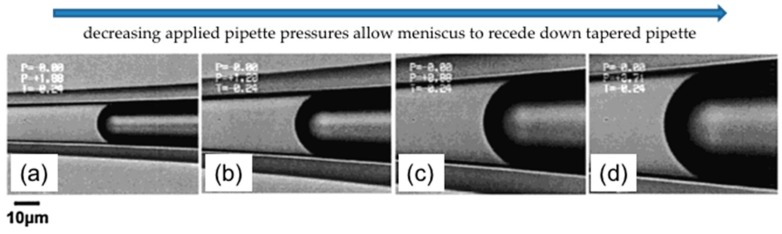
Microscope images of an air-water surface inside a tapered micropipette. (**a**) The air-filled micropipette was inserted into the surfactant solution under relatively high positive applied pipette pressure 18.8 kPa. The meniscus came to equilibrium such that the interface meniscus (diameter ~15 µm) was close to pipette tip. (**b**–**d**) The applied positive pressure was then decreased to, (**b**) 12 kPa, (**c**) 8.8 kPa, and (**d**) 7.1 kPa respectively allowing the meniscus to recede down the tapered pipette to new equilibrium curvatures. In the experiment this is followed by a series of increasing pressures that move the meniscus back down the pipette and so is advancing. There was no hysteresis in the positions or contact angle for these receding or advancing contact angles. With permission from American Chemical Society [9].

The simplest measurement that can be made using the micropipette technique is to validate the well-established clean air-water surface tension using the tapered micropipette [9]. (This is actually an experiment we use to train new researchers on the micropipette manipulation system). Figure 5 shows a series of typical air-water surface images inside a micropipette corresponding to four different applied positive Laplace pressures [9].

**Figure 6:**
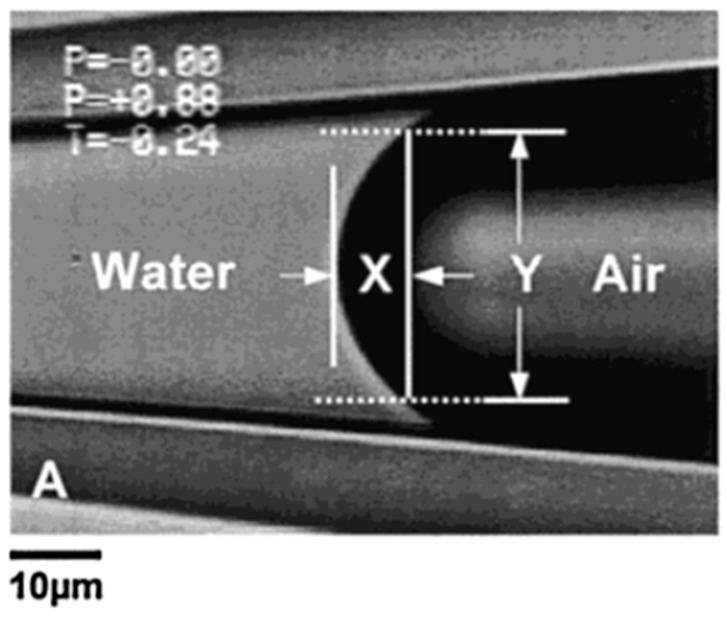
Calculation of radius of curvature *R_c_* from the geometry of the surface image. With permission from American Chemical Society [9].

As described previously (Section 2.1., Principles of Capillary Action and the Micropipette), water enters and flows continually into a horizontal micropipette by capillary action if we do not apply any positive, opposing pressure inside the pipette. In the experiment the micropipette was inserted into the surfactant solution under an initially relatively high pre-set positive applied pipette pressure of 18.8 kPa. As seen in Figure 5a, the meniscus came to equilibrium with a diameter of ~15 µm such that it was close to pipette tip. A subsequent decrease of the applied pressure from 18.8 kPa (Figure 5a) to 7.1 kPa (Figure 5d), resulted in the movement of the air-water meniscus to larger and larger radii in the tapered pipette. This control of the meniscus position (and hence radius of curvature of the interface) inside the tapered micropipette, provides the surface tension measurement by simple application of the Laplace equation, Equation (5). Figure 6 shows how to calculate the radius of curvature *R_c_*, from the air-water surface geometry, defined by the vertical distance (*Y*) and the horizontal distance (*X*) that correspond, respectively, to the chord between the ends of an arc spanning the cap and the height of the cap. 

From the geometry of the interface inside the capillary, the following relation is obtained,

(6) $$R_{c}=\frac{{\left(\frac{\gamma }{2}\right)}^{2}+X^{2}}{2X}.$$

**Figure 7:**
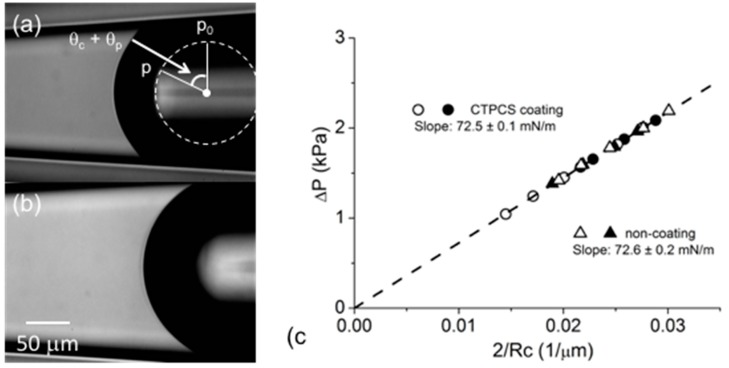
Contact angle change with and without silane coating at the glass surface of the micropipette at 20 °C and corresponding surface tension plot. (**a**) 3-Cyanopropyltrichloro silane (CTPCS) coated micropipette, having a contact angle of 54° and applied pressure of ~1.4 kPa inside the pipette. Using the fitting of curvature (dashed circle) at the diffraction pattern at the air surface area, the contact angle is calculated by considering the point “p” and “p_0_” giving 54 ± 7°. Note this circle is not at the interface but at the point at which the meniscus leaves the glass surface. Point “p” shows the grey and black pattern boundary spot on the circle, and p_0_ shows a crossing point of the circle and a vertical line crossing the circle centre. (**b**) Non-silane coated (just glass) micropipette having ~1.4 kPa applied pressure inside the pipette with the much smaller contact angle of 5°. (**c**) Equilibrium air-water surface tension measured by the micropipette manipulation method with increasing pressure (hollow symbols: interfacial area decreasing) and decreasing pressure (solid symbols: interfacial area increasing) comparing with and without CTPCS coating. The surface tension was calculated from the slope of a plot of applied pressure Δ*P* vs. the reciprocal radius of curvature 2/*R_c_* at each applied pressure. With permission from Elsevier [7].

As mentioned above, the tapered micropipette manipulation technique solved the *θ_c_* problem by using *R_c_* in the equation rather than using *R*, thus not requiring any factor of *θ_c_* at the point where the three phases (air-water-glass) meet. In order to check this, we measured the air-water surface tension for two different contact angles. A glass pipette surface coating of 3-Cyanopropyltrichloro silane (CTPCS) produced a much higher contact angle of 54° compared to that for a clean glass surface of 5° [7]. Figure 7a (CTPCS-coated) and Figure 7b (non-silane coated) show the images of the air-water surface with different contact angles at ~1.4 kPa applied pressure.

From the fitting circle at the edge of the diffraction pattern (Figure 7a, white dashed circle), the contact angle *θ_c_* of the air-water surface against the hydrophobic CTPCS-coated micropipette glass surface was estimated to be *θ_c_* = 54 ± 7°. This showed good agreement with other literature data of 56.3 ± 2.2° [89]. We therefore confirmed that the estimation of *θ_c_* from the diffraction pattern was a reliable method for the air-water surface tension measurement. By comparison, the contact angle against the clean glass surface of 5°, shown in Figure 7b is, as expected, much smaller than the CTPCS-coated micropipette. Satisfyingly, in Figure 7c, both measurements of Δ*P* vs. 2/*R_c_* plotted for a series of different applied Δ*P* showed the same linear fitting slope. Then, using Young–Laplace, Equation (5), the slope expressed the *γ* values, and these were, *γ* = 72.5 ± 0.1 (CTPCS coating) and 72.6 ± 0.2 mN/m (non-coating), at 20 °C. Thus, even though the two glass surfaces were of completely different hydrophobic or hydrophilic character, the measurement of the clean air-water surface tension was self-consistent in excellent and accurate agreement with reference data from the literature [90]. 

What these simple experiments show is that the radius of curvature *R_c_* is accurately determined from a segment of the interface and the contact angle is not required in order to measure the surface tension with the tapered micropipette system.

### 2.4. Liquid-Liquid Interfaces

**Figure 8:**
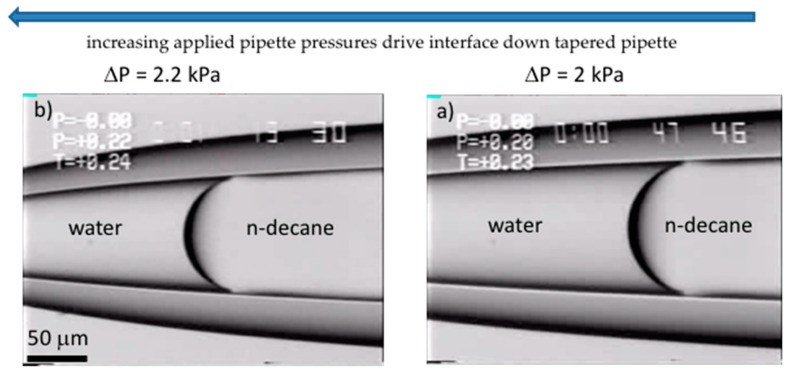
Equilibrium interfacial tension for the simple oil-water system. Shown are video-micrographs from an experiment where n-decane filled the micropipette and water was placed in the microchamber. (**a**) (right-hand micrograph) As in Figure 5, the (partially) oil-filled micropipette was inserted into the water under positive applied pipette pressure Δ*P* of 2 kPa to give an interface meniscus in the pipette taper with a diameter ~100 µm. The applied positive pressure was increased to (**b**) 2.2 kPa, and the oil-water interface was moved to a new position with a smaller diameter of 93 µm. This was repeated to obtain the advancing and receding meniscus positions and corresponding radii used to plot the data in Figure 9.

We have also established the technique for making equilibrium interfacial tension measurements at oil-water interfaces [9]. By partially filling the pipette with oil and placing water in the microchamber, a curved interface can be formed in the micropipette, much like the one between air and water. Since oil is hydrophobic the curvature of the meniscus in contact with the glass pipette surface is again dependent on water-wetting of the hydrophilic glass. As with the simple air-water system, as shown in Figure 8, the application of positive pipette pressure moves the interface position down the pipette (Figure 8a,b) changing its radius. The application of negative pressures then allows it to recede back up the pipette for reversible measurement of advancing and receding positions.

**Figure 9:**
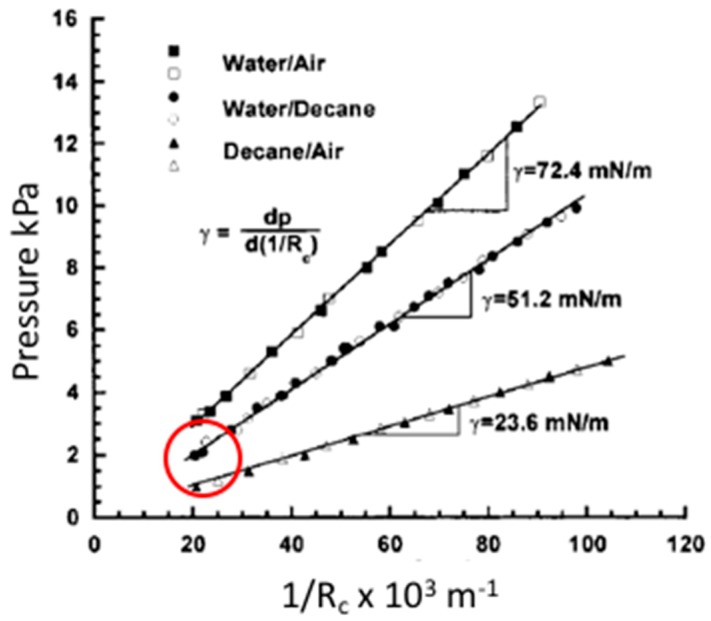
Plot of Equilibrium surface and interfacial tensions measured by the tapered micropipette manipulation technique. Data for applied micropipette pressure Δ*P* (kPa) is plotted in this particular paper [9] against the reciprocal of the radius of curvature 1/*Rc* (×10^3^ m^−1^) for Water/Air, Water/Decane and Decane/Air. Meniscus position was measured for both increasing pressure (hollow symbols) and decreasing pressure (solid symbols). As before (Figure 7), the surface tension was calculated from the slope of the plot. With permission from Elsevier [9]. The circled data correspond to the menisci shown in Figure 8, i.e., at the lowest pressures and largest radii of curvature.

As before (Figure 5, Figure 6 and Figure 7 and associated text), this allows the interfacial tension to be measured using the Laplace equation, Equation (5). From the best fitting slope to a plot in Figure 9 of applied pressure Δ*P* vs. 2/*R_c_* at each applied pressure, gives the interfacial tension for the decane-water interface as 51.2 ± 0.4 mN/m [9]. Additionally shown on the plot is plot and surface tension for the decane-air interface as 23.6 ± 0.5 mN/m [9]. These values were in good reasonable agreement with literature values [91]. In general, the oil-air interfacial tension has a lower value compared with the oil-water interfacial tension. For completion, and to summarise this section on gas-liquid and liquid-liquid systems, Table 1 shows the list of different alkane or alkanol interfacial and surface tensions against water γ_OW_ or air γ_OA_. The results from the tapered micropipette manipulation technique (bold letters) are combined with those from the literature [91,92]. Interestingly, for the completely non-polar alkanes the alkane-water surface tension (~51 mN/m) is always larger than the alkane-air tension (~21 mN/m). The alkanol-air tensions (~27 mN/m) are similar to those of the alkane-air due to the presentation of their own alkane chains to air. Conversely, at the alkanol-water interface, the polar head group of the alkanol can orient towards the water (see later, Figure 11a) and provides a lower interfacial tension (~8 mN/m) than to air. This result clearly suggests that alkanols have a monolayer-forming capacity and can act as surface active compounds, an effect that we examined for 1-Octanol [7] and present and discuss later as equilibrium and dynamic adsorption (see Section 4. Equilibrium and Dynamic Surface Tension: Adsorption of Soluble Surfactants). As mentioned at the beginning of this review, a pure hydrocarbon chain molecule, like decane cannot spread at the air-water surface because of a lack of molecule polarity [21]. 

In order to finish this section on the development of the micropipette techniques, we present now our most recent Micropipette Interfacial Area-expansion Method (MIAM) that has allowed us to make Dynamic Surface Tension (DST) measurements.

### 2.5. More Advanced Techniques: Micropipette Interfacial Area-Expansion Method (MIAM) 

The initial tapered micropipette technique was developed and introduced by Lee et al. in 2001 [6,9]. It was used to measure equilibrium tensions of clean and surfactant-adsorbed surfaces and also to make dynamic surface tension measurements for the adsorption of phospholipids (as described later, see Section 5. Equilibrium and Dynamic Surface Tension: Adsorption of Insoluble Surfactants). The system was able to track dynamic surface tension changes for times on the order of ~15 s. It was successful in that it demonstrated the formation of lipid monolayers from adsorbing lipid vesicles in the aqueous phase, that came to equilibrium in times on the order of 2 min. While successful for lipid adsorption, it clearly had limits for faster-transport-surfactants. In the dynamic process of lipid or surfactant adsorption to a clean surface or interface, there is a decrease in tension and so a meniscus-interface in the tapered micropipette would move to smaller radii for a constant applied pipette pressure. While we did manage to blow surfactant solution at a clean interface using a smaller delivery pipette inserted into the larger tapered pipette (see Figure 6 and Figure 7 in Lee et al. [9]) and observe the rapid movement of the meniscus to correspondingly smaller radii, the limits of this early technique were simply due to not being able to move the micropipette inside the chamber fast enough to observe the meniscus position (and measure its radius). Therefore, for more advanced applications (see later in Section 6 Applications) and indeed for more accurate and sensitive fundamental studies in dynamic tension measurement for surfactants, we developed the Micropipette Interfacial Area-expansion Method (MIAM) [7]. 

**Figure 10:**
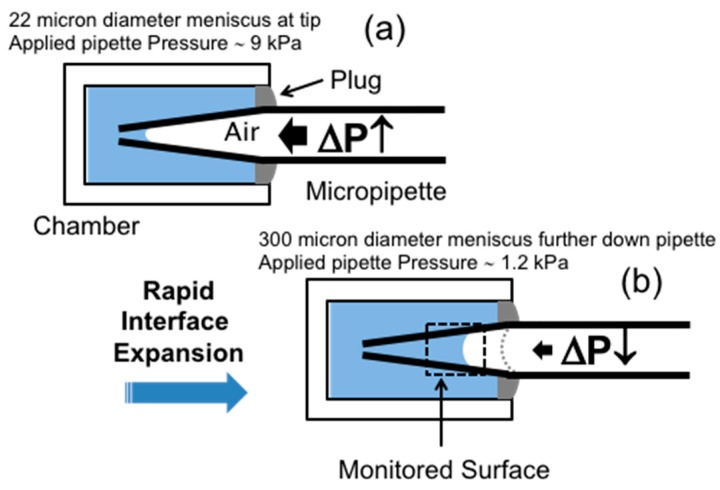
Schematic image of the Dynamic Surface Tension (DST) measurement using the Micropipette Interfacial Area-expansion Method. (**a**) The micropipette is inserted into the surfactant solution under high positive applied pipette pressure such that the new interface meniscus (diameter ~22 µm) is close to the pipette tip. This is observed by initially positioning the micropipette tip in the field of view. To avoid convective flow inside the chamber, the microchamber is gently sealed with a plug of hexadecane after the pipette is inserted and the pipette moved to a new viewing position where a clean surface is expected to locate when a pre-set lower pipette pressure is applied. (**b**) Following a 15-fold decrease of the applied pipette pressure, Δ*P*, (from the high value of ~9 kPa, that held the interface close to the pipette tip, to 0.6 kPa), the surface area quickly expands to a new diameter of ~300 μm in 0.1 to 0.3 s. The pipette pressure is rapidly fixed to a constant reset pressure of 1.2 kPa, and the surface meniscus moves to the observed region (dotted box). The movement of the surface meniscus is tracked as surfactant adsorbs to the water-air surface and reduces the tension to the new equilibrium. With permission from Elsevier [7].

In order to improve the time-lag, any need for micropipette movement was eliminated by fixing the micropipette a priori at a position that represented the expected meniscus diameter for a clean air-water surface (i.e., ~72 mN/m tension). This surface was then ready for the initial adsorption and therefore setting a diameter for time zero [7,8]. Figure 10 shows schematic images of the Micropipette Interfacial Area-expansion Method as used in this technique and a description of the sequence of events. 

The method follows a protocol of these three steps:

Step 1: Set the position of the micropipette inside the microchamber and apply a high positive pressure (~9 kPa) by using a syringe pump. This pressure minimises the air-liquid meniscus area and makes it almost coincident with the micropipette tip (radius ~11 μm) (Figure 10a). The end of the microchamber is sealed with a small volume of hexadecane to avoid water evaporation and limit convective flow during the experiment. 

Step 2: Quickly release the applied pressure from 9 to 0 kPa in 0.1 to 0.3 s. This achieves a 200-fold interfacial area expansion, i.e., from 700 to 140,000 μm^2^, as shown in Figure 10b, and moves the meniscus to the position indicated by the grey-dashed meniscus in the pipette. This rapid surface area expansion creates an essentially clean air-water surface in the micropipette (i.e., with only ~0.5% of the initial concentration of the surfactant that would have been adsorbed at the initial small interface). 

Step 3: After the rapid expansion of the surface area, quickly apply a reset pressure ~1.0 kPa, observe the position of the surface meniscus (dotted box) and proceed with the *R_c_* measurement of its gradual movement down the micropipette taper to smaller radii at this constant applied pipette pressure. That is, the maximum surface area becomes smaller when surface active agents adsorb at the relatively clean air-water surface (Figure 10b, monitored area). Thus, the dynamic change of the position of the meniscus in the tapered micropipette and hence its corresponding surface curvature is monitored and recorded in real time for later analysis. 

This technique then was developed to capture the fastest times possible for surface and interfacial movements in response to surfactant adsorption. Of course, this process includes the diffusion of surfactants to (and from) the surface and so embedded in it, and obtainable from the results, is the diffusion coefficient of the adsorbing species. All this will be presented and shown later for both non-ionic and ionic test surfactants (Section 4 and Section 5) and for an application studying lung surfactants (Section 6.3.). First, though, we consider the entities involved in the equilibrium and dynamic surfactancy that we have studied.

## 3. Entities: Soluble and Insoluble Surfactants as Monolayers, Micelles, Vesicles and Emulsions

**Figure 11:**
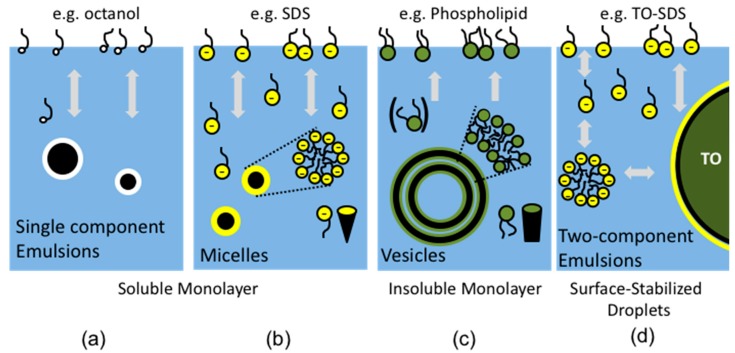
Surface active compounds at the air-water surface and in bulk aqueous phase. (**a**) Soluble Monolayer: Long-chain alcohols, such as Octanol, can spread at the air-water (blue background) surface to make a soluble monolayer (monomer can transfer between monolayer and bulk). The monolayer has a certain orientation, i.e., hydrophilic OH-headgroup (white circle) towards the water and hydrophobic C8 alkyl chain (black curvy line) towards the air. Above its solubility limit in bulk aqueous solution, octanol can behave like pure hydrophobic oil, i.e., forming its own oil emulsion with octanol also adsorbed at this interface. (**b**) Soluble Monolayer: Detergents, such as the anionic sodium dodecyl sulphate (SDS), can spread at the air-water surface to form a soluble monolayer. The orientation is the same as the long-chain alcohol with the negatively charged hydrophilic polar group facing the aqueous phase, (yellow circle) and the hydrophobic C12 alkyl chain towards the air (black curvy line). Above its solubility limit in bulk, monomers form micelles. (**c**) Insoluble Monolayer: Lipids, such as phospholipids, can spread at the air-water surface to form an insoluble monolayer, again with headgroups in the water phase (green circles) and double acyl chains in air (double black curvy lines), with relatively little molecular lipid (bracketed) in solution (water solubility, *S_w_* ~10 nM or less). In the bulk aqueous phase monomer lipids self-assemble into vesicles. (**d**) Surface-Stabilised-Droplets. Micro- or nano-emulsion droplets can be stabilised with, for example, the anionic detergent SDS.

By way of a more technical introduction to the systems we have studied, we now briefly describe the surfactant- and lipid-systems in which our micropipette manipulation experimentation has made some important contributions. Amphiphilic compounds are well-known surface-active agents at the air-water surface and also form association colloids. The schematic images in Figure 11 show typical behaviours for some common examples of these compounds at the air-water surface as soluble monolayers and in bulk solution as micelles, as well as insoluble monolayers and membrane vesicles of the relatively more-insoluble lipids. In our earlier micropipette work in 2001, we established the rudimentary methods for measuring equilibrium tensions of clean and surfactant-adsorbed surfaces [9] as well as the equilibrium and dynamic tensions due to adsorption of phospholipids [6]. In 2017 we have now improved on, and extended, these techniques for both equilibrium and shorter-time dynamic behaviour of the surface-active agents Octanol [7] and sodium dodecyl sulphate [8], and as described later in Section 6.3., for lung surfactant formulations [10].

Simple amphiphilic compounds can create monolayers at the air-water surface by orienting their monomers, i.e., hydrophilic polar head groups towards the water and hydrophobic chain(s) towards the air. Upon formation of a monolayer of these compounds, the air-water surface tension is decreased from 72 mN/m (at 20 °C) to much lower values depending on the surface concentration (Γ), and characteristics of these compounds, but can be on the order of 30 mN/m. As depicted in Figure 11, amphiphilic compound-monolayers can be separated into two groups, i.e., soluble and insoluble monolayers [93]. When monomers of the monolayer have significant solubility in the aqueous phase and can exchange with excess monomers in the equilibrium bulk solution, it is called a soluble monolayer. These monomers rapidly come to equilibrium and maintain a relatively constant surface pressure due to rapid desorption or adsorption when compressed or expanded, respectively. If the monomers are relatively insoluble in water and remain at the surface rather than exchanging during any monolayer expansion or compression, it is called an insoluble monolayer. These different characteristics of monomers can also result in different aggregation behaviour in bulk aqueous media. For example, soluble surfactants tend to form micelles (Figure 11b) that can exchange with soluble monolayers, while lipids form bilayer-membrane vesicles (Figure 11c) that can form insoluble monolayers but are less likely to exhibit rapid exchange. In all cases, above the solubility limit of the monomers, they coexist with these self-assembled-aggregates in the bulk water. Interestingly, the surfactants themselves, e.g., octanol, even in the absence of a second oil phase, can form their own “oil droplets” as microscopic emulsions and as nanoparticles in aqueous suspension (as shown in Figure 11c). Next, we consider the physicochemical characteristics of each amphiphilic compound and how it influences the micropipette manipulation-measurements we have made. 

### 3.1. Long-Chain Alcohols form Soluble Monolayers and Emulsions

**Figure 12:**
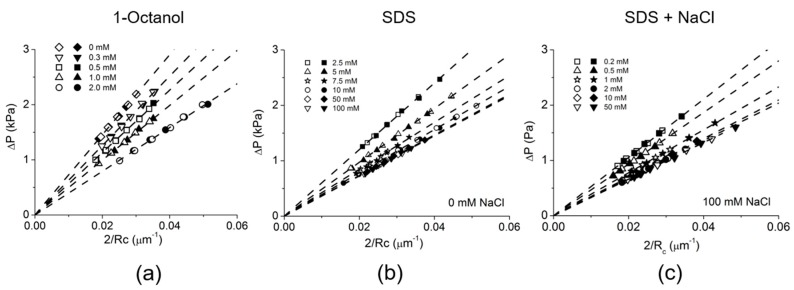
Equilibrium surface tension measurements for soluble surfactants. Pipette pressure vs. 2/*R_c_* plots for test surfactants Octanol and SDS to give equilibrium surface tension of soluble monolayers at each of the test concentrations in aqueous solution for (**a**) 1-Octanol, (**b**) SDS and (**c**) SDS with 100 mM NaCl. Measurements were made for both increasing and decreasing applied pressure at 20 °C. Hollow and solid symbols show the direction of applied pressure—hollow symbols: pressure increasing, interfacial area decreasing; and solid symbols: pressure decreasing, interfacial area increasing. Each surface tension was calculated from fitting slope as mentioned in the text corresponding to the plots in Figure 7c. Reproduced from Kinoshita et al. [7,8], with permission from Elsevier.

**Figure 13:**
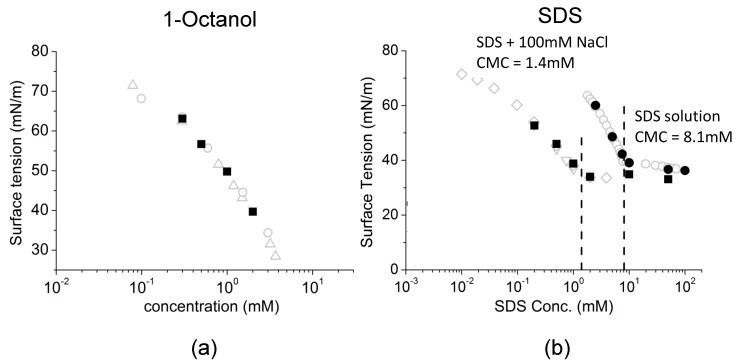
Equilibrium surface tension vs. soluble surface active compound concentrations measured by the tapered micropipette manipulation technique at 20 °C. (**a**) 1-Octanol equilibrium surface tension made by the micropipette technique (solid squares). The obtained data was compared to literature values, which were measured by Du Noüy’s ring (open triangles) and Pulsating Bubble Method (PBS) methods (open circles) [130,131]. (**b**) SDS equilibrium surface tension in the absence (solid circles) and presence (solid squares) of 100 mM NaCl again made by the micropipette technique [7,8]. Literature results from the Wilhelmy plate method (open circles), drop volume (open diamonds) and maximum bubble method (inverted triangles) are also added for comparison [25,132,133]. The dashed lines show the critical micelle concentration (CMC) values, 8.1 mM (pure water) and 1.4 mM (100 mM NaCl), which agree with literature [9,25,98]. Reproduced from Kinoshita et al. [7,8], with permission from Elsevier.

In general, water-miscible, short-chain alcohols decrease the surface tension by forming a monolayer at the air-water surface. For example, by increasing the concentration of the water-miscible solvents methanol, ethanol, and propanol in an aqueous solution, there is a concomitant decrease in the air-water surface tension from 71 to ~ 23 mN/m at 20 °C [94]. In the case of the longer-chain, water-immiscible alcohols, such as 1-Octanol (see below, Section 4.1.) they can also decrease the air-water surface tension to ~25–30 mN/m [7], by forming a soluble monolayer at the surface, and so not quite as much as for short-chain alcohols. A special case for longer-chain alcohols is their ability to form emulsion droplets (Figure 11a) and hence an immiscible interface. Interestingly, the long-chain alcohol molecules start aggregating into emulsion droplets before reaching the lowest surface tension. This behaviour correlates with the solubility of the carbon chain, i.e., the longer the carbon chain length, the lower its solubility in water [21] and the greater its tendency to form the emulsion droplet. As mentioned in the Introduction 1.1, non-polar, pure hydrocarbon chains, such as the n-alkane homologous series, cannot spread as monomolecular films at the air-water surface. Therefore, just the addition of one terminal-OH group converts the relatively insoluble and surface inactive octane to a more soluble and surface-active octanol. As described in more detail below, (Figure 12 and Figure 13) our micropipette experiments have measured the rapid and dynamic adsorption of Octanol to otherwise clean air water interfaces and shown their rapid approach to equilibrium tensions [7]. These measurements provided a value for the diffusion coefficient of 1-Octanol, of 7.2 ± 0.8 × 10^−6^ cm^2^/s. While highly related, but beyond the main scope of this review, once we obtained a microdroplet of octanol in water, the micropipette technique was further developed (along the lines as for gas bubble dissolution, see Section 6.1.1.) to measure the dissolution of octanol [7,16] into water, as well as a series of organics and their mixtures [95]. Briefly, the dissolution of a 50 μm diameter microdroplet of octanol into water takes a relatively long time, ~3000 s. This is because, as we can see according to the Epstein Plesset equation (see later Equation (8) [96]), the rate of loss of material, dm/dt, is a function of the product of the diffusion coefficient D (of octanol in water) and its water-saturation concentration *C_s_*. In contrast to a dissolving gas bubble (Section 6.1.1.) that has a finite surface tension and hence a Laplace pressure, for a liquid microdroplet the interfacial tension and the Laplace pressure play virtually no role because of the incompressibility of the octanol. Thus, the very low solubility of octanol (and other organics) in water increases their microdroplet dissolution time. This experiment has thus allowed us to actually measure the diffusion coefficients for octanol to be 7.3 ± 0.1 × 10^−6^ cm^2^/s, in excellent agreement with the result from the dynamic adsorption technique [7]. Dissolution is also proportional to 1/*R* and so large droplets on the order of millimetres might appear to “never” dissolve. This is another key aspect of being able to directly observe and measure microdroplet behaviour with the micropipette technique and our single microparticle micropipette experiments, i.e., for micron-scale dimensions (e.g., *x* = 100 μm) and diffusion coefficients on the order of 5 × 10^−6^ cm^2^/s, Einstein’s mean-square displacement diffusion equation [97], *x*^2^ = 2*Dt*, puts the time *t* = 10 s, whereas for 10 millimetre-sized particles, t is on the order of 100,000 s (~28 h). Thus, because of the scale of our experimentation, the results of micropipette manipulation experiments are observable in our laboratory time frame.

### 3.2 Soluble Monolayer: Detergents form Micelles

More common than alcohols are the well-known ionic surfactants such as sodium dodecyl sulphate (SDS). This anionic detergent can decrease the air-water surface tension from 71 mN/m to around 40 mN/m by forming a soluble monolayer at the air-water surface (see Section 4.1.) [8]. One of characteristics of this compound is that it readily forms micelles at its limit of monomer-solubility (8.1 mM) in bulk water (Figure 11b)—its critical micelle concentration, CMC. (Note, this micelle is also in solution and has its own dynamic, solubilised interface). Compared with the self-assembly of long-chain alcohols into actual emulsion droplets, SDS forms micelles in water where 30 to 70 monomers are oriented per micelle depending on concentrations in excess of the CMC [98,99]. Interestingly, in NaCl solution, the aggregation number is increased to ~130 (in 0.4 M NaCl) because of electrostatic shielding of the sulphate negative charge so allowing a more close-packed arrangement of the sulphate headgroups. As described in more detail below (Figure 12 and Figure 13), using similar techniques as developed for octanol, our micropipette experiments have also measured the rapid and dynamic adsorption of SDS to otherwise clean air-water interfaces and have shown their rapid approach to equilibrium tensions [8]. 

### 3.3 Insoluble Monolayer: Lipids form Vesicles

The most well-known insoluble monolayer is the phospholipid monolayer. Actually, the study of insoluble monolayers was first established by studying fatty acids [100]. Unlike the rapidly-exchanging, soluble monolayers, insoluble monolayers are characterised by complex 2-dimensional phase behaviour as surface pressure-molecular area (Π-A) diagram, nicely reviewed by [101]. Such pressure vs. area curves characteristically include two-dimensional analogues of solid, liquid, and gaseous states along with intermediate phases such as the liquid-expanded and liquid-condensed regions appearing on occasion between the main states [102]. The addition of a small amount of a phospholipid to a clean air water interface results in a rapidly spread monolayer of the material and, again, a reduction in surface tension to values that can be ~20–25 mN/m at the air-water surface (again measured by our micropipette technique [10], see later Section 6.3. Lung Surfactants) and as low as 1–2 mN/m at the oil-water interface (see Section 6.4. Nanoprecipitation) [17]. 

The unique measure of lipid self-association is characterised by the Critical Bilayer Concentration (CBC). In general, the “CBC” of such insoluble monolayer compounds is extremely low compared with soluble monolayer compounds such as SDS (CMC 8.1 mM). For example, the CBC for dipalmitoyl-PC (DPPC, two carbon chains of C16:0) is 0.46 nM [103]—seven orders of magnitude smaller than the CMC of SDS! This is because of the greater hydrophobicity of DPPC’s two long hydrocarbon chains compared to SDS’s one. These lipids form vesicles as shown in Figure 11c, where simple rehydration of dried lipid can form multi-lamellar vesicles (MLV) having bilayer membranes in a sphere shell shape [104,105,106]. Vesicles can also be made as small (100 nm) unilamellar vesicles (SUVs) with special extrusion processing [107] or can be large enough as so called Giant Unilamellar Vesicles (GUVs) to make microscale measurements on them with the micropipette by gentle rehydration. The mechanochemistry, thermal and interactive properties of these GUVs also have been studied extensively by the micropipette technique as reviewed many times [73,80,108], and also recently by Parra and Needham [109]. 

Interestingly, since air is relatively hydrophobic, a similar phenomenon occurs at the oil-water interface and a spread monolayer is formed. As established by Mingins [110], such materials can also be described by isotherms of surface pressure (π) against area per molecule (A) reported for a homologous series of pure synthetic saturated 1,2-di-acyl glycerophosphocholines (lecithins) (C14 to C2,) spread at n-heptane/aqueous electrolyte interfaces. Haydon also measured lipid spreading on monolayers and the surface-potential changes in lipid monolayers and the ‘cut-off’ in anaesthetic effects of N-alkanols [111]. Therefore, here we have a situation where a phospholipid monolayer is now a solvent for n-alkanols, and is actually an interesting test system for anaesthetic absorption into bilayer membranes and the proteins they contain in nerve cells. In another anomaly, as presented in the introduction, a single chain surfactant like Glycerol MonoOleate (GMO) can actually form bilayers if its molecular volume is enhanced with adsorbed alkanes, and so forms the so called “black lipid films” [39,112]. As described above (Section 1.3) these bilayer films [47] have been characterised in terms of free-energies of formation from solvent-containing to solvent free, [1,38] and used extensively as model biological membranes, for anaesthetic adsorption and channel formation and activity [113,114]. 

Thus, lipid monolayers have been studied extensively in their own right, and interestingly, when literally pushed to their limit, i.e., when the monolayer is compressed above a certain surface pressure limit, they collapse into vesicles rather than reverting to monomers [115,116]. They can even exist in equilibrium with vesicles that are placed in the aqueous sub-phase, as shown by MacDonald and Simon [117]. Here, the collapse pressure was similar to the internal pressure of lipid bilayers (~50 mN/m), which corresponds to a true equilibrium for unstressed liposomes. Such monolayers of DMPC also underwent the same phase transitions as would a vesicle. Their data agreed well with Needham and Evan’s data [76] on the same DMPC lipid as a GUV. Thus, the mechanical and thermodynamic properties of bilayers, particularly phase-transition parameters, have corresponded closely to those of monolayers with which they are in equilibrium. In the context of lipid bilayer vesicles, while we are mainly focused here on surface and interfacial tensions at interfaces, for completion, the micropipette technique has been also used extensively to apply well defined tensions and measure the mechanical [73,78,80,83,108,109,118], thermal [72,76,77], molecular exchange and inter-bilayer interaction properties [73,119] of giant (20–30 μm-diameter) phospholipid vesicles [120].

Returning to lipid monolayers, some of our first measurements were made while developing and using the micropipette technique for measuring the dynamic and equilibrium surface tensions of adsorbed phospholipid monolayers from aqueous suspensions of uni-lamellar lipid vesicles to clean air water interfaces [6,9] (see later Section 5, Adsorption of Insoluble Surfactants). These were followed 18 years later by using the improved micropipette methods to observe and measure the adsorption and complex multi-bilayer forming mechanism of lung surfactants, seen earlier by others [10,121,122,123,124,125]. 

Thus, it is generally accepted that phospholipids form bilayers and soluble surfactants form micelles. However, when the carbon chains of the phospholipids are made extremely short, these compounds can also make micelles rather than bilayer vesicles [100]. Such short chain phosphatidyl-cholines (PC) are structurally phospholipids, but their short fatty acyl chains of 6–8 C-atoms endow the molecule with detergent-like properties [126]. Since surfactants are usually single chain and the lipids are usually double chain, there is a relationship between the molecular volume, its headgroup area at the interface and the length of the hydrocarbon chain [93], and so micelle- or vesicle-formation can be described by a packing parameter model [127].

### 3.4. Surfactants Can Adsorb at Oil-Water Interfaces and the Oil Can Swell Micelles

Finally, just as they can adsorb at air-water surfaces, surfactants can adsorb at oil-water interfaces. Air is hydrophobic, and so just like at the air water interface, the hydrocarbon chains of a surfactant partition in the same way at oil-water interfaces. As shown by example, in Figure 11d, micro- or nano-emulsion droplets of Triolein can be stabilised with the anionic detergent SDS. For this situation, several equilibria are set up: SDS molecules in solution are in equilibrium with the monolayer at the air-water surface, with micelles if above the CMC, and with the oil-water interface of the emulsion. Interestingly, the micelle is also in equilibrium with the bulk phase of the emulsion, and can contain a number of oil molecules that are in equilibrium with the oil in the bulk phase. In unpublished studies (Needham, undergraduate projects) we hypothesized in 2000, that this represents just enough molecules to create bulk matter [128]. We found that, indeed, small alkanes (pentane to decane) would swell micelles of Tween 20 (hydrogen-saturated C12 monolaurate chain) showing a statistically significant increase in hydrodynamic radius within the first 48 h from the initial micelle radius of 4.42 ± 0.08 nm to 6.5 ± 0.09 nm for the pentane system, to 7.84 ± 0.33 nm for the decane system. If the radius of the micelle is equivalent to the size of the Tween20 molecule, and we assume that this represents the thickness dimension of the Tween80 monolayer on the swollen micelle, then the alkane-core-radius for decane is simply 7.84 nm − 4.42 nm = 3.42 nm, which gives a core-volume of decane of ~168 nm^3^. Since decane has a molar volume of 195 cm^3^/mol, and so volume per molecule of 0.32 nm^3^/molecule, the micelle core would contain 525 molecules. Here the molecules in the swollen micelle are expected to be in equilibrium with, and so at the same chemical potential as, the bulk emulsion. Is this how many molecules of decane it takes to create bulk matter? On-going experiments in our lab are now exploring this further.

As is often said by our friend and collaborator Evan Evans, there are only two things we need to know in characterising any system scientifically, “where it is going” (equilibrium thermodynamics) and “how long it takes to get there” (kinetics or dynamics). Therefore, following this maxim, next, we give examples of using these techniques for equilibrium and dynamic surface tension measurements on soluble surface active materials (Octanol and SDS) and insoluble phospholipids (the Phosphatidylcholines). 

## 4. Equilibrium and Dynamic Surface Tension: Adsorption of Soluble Surfactants

In 2017, we presented new measurements of the equilibrium and dynamic surface tensions of soluble monolayers of 1-Octanol and SDS at the air-water surface using the tapered micropipette manipulation technique [7,8]. Since SDS is anionic, we would expect shielding of the electrostatic interactions between molecules at the surface for SDS in NaCl, reflected in a lower CMC. Experimental conditions therefore included milli-pure water and high ionic strength NaCl solutions in order to evaluate the effect of charge of the SDS molecule on adsorption rates and monolayer formation. It was these measurements that necessitated the development of the more advanced Micropipette Interfacial Area-expansion Method (MIAM) (see previous Section 2.5.) [7,8].

### 4.1. Equilibrium Surface Tension for Adsorption of Soluble Surfactants: Octanol and Sodium Dodecyl Sulfate

As mentioned above (Figure 11a) [129], the long-chain alcohol, 1-Octanol has a limiting solubility of *C_s_* = 3.53 mM in aqueous media. Above this solubility limit concentration, its molecules can self-assemble as oil emulsions. Therefore surface tension measurements were done at much lower solution concentrations than the *C_s_* of 1-Octanol i.e., 0, 0.3, 0.5, 1.0 and 2.0 mM [7]. Figure 12a shows plots of the applied micropipette pressure ΔP vs. the reciprocal radius of curvature as 2/*R_c_* for the air-water meniscus in the pipette at each applied pressure for each 1-Octanol solution concentration.

The equilibrium surface tension was calculated from the best fitting linear slope of the plots at each concentration. As we saw for the clean air-water surface tension in Figure 7c, the linear slope at each concentration did not show any significant difference between advancing, and so shrinking the area of the meniscus (hollow symbols) and receding, and so expanding the area of the meniscus (solid symbols) with applied pressure control. This clearly demonstrates that the transfer of octanol monomers between the monolayer and in solution (Figure 11a) is rapidly reversible showing slow smoothly-performed area change at the air-water surface under applied pressure control. As shown in Figure 11b, SDS can make micelles above its CMC of 8 mM in water or 1.4 mM in 100 mM NaCl. Thus, SDS measurements were made in Milli-Q-purified water and in 100 mM NaCl (Figure 12b,c) and the obtained radius of curvature values at each pressure were again plotted in the 2/*R_c_* vs. ΔP plot at each SDS concentration and found to be linear. 

As expected and shown in Figure 13a the octanol data showed a linear relationship between surface tension and aqueous concentration of Octanol (on a logarithmic scale), consistent with previously published data measured by Du Noüy ring and Pulsating Bubble Method (PBS) methods [130,131]. As also expected for SDS, below its CMC the plots showed a similar linear behaviour between surface tension and log concentration and then levelled out as the CMC was approached and exceeded. For SDS, in Figure 13b, the effect of charge shielding in the presence of NaCl shifted the curve to a lower concentration range consistent with its lower CMC. In fact, the change in slope for surface tension vs. concentration, is actually used to define the CMC and gives CMC values of 8.1 mM for SDS in pure water and 1.4 mM for SDS in 100 mM NaCl (Figure 13b, dashed lines).

Thus, Octanol shows a monotonic decrease in surface tension with increasing log of octanol concentration up to its solubility limit. For SDS, when micelles form they compete with the monolayer and the surface adsorption is limited and actually produces a change in slope for surface tension vs. concentration. Above the CMC, SDS monomers still continue to adsorb at the air-water surface as shown by the slightly decreasing surface tension. As a prelude to making dynamic tension measurements, these systems allowed us to validate that the tapered micropipette manipulation technique was capable of making accurate and reproducible measurements of soluble monolayer equilibrium surface tensions [7,8]. 

### 4.2. Dynamic Surface Tension for Adsorption of Soluble Surfactants

One of the biggest challenges of interfacial tension measurements is how to measure a precise dynamic surface tension change in such short times that are associated with molecular diffusion to the surface [134]. Until now, only a few techniques have been successful and achieved reliable data that could be analysed in terms of theoretical models. For technical reasons, the majority of interfacial tension measurement techniques were not fast and/or sensitive enough to detect dynamic surface tension changes during, for example, initial surfactant adsorption to a clean interface and de novo monolayer formation. In order to bring a new technique to the field and see how far we could take this micropipette technique, in 2017 we developed and the tapered pipette methodology Micropipette Interfacial Area-expansion Method (MIAM) [7,8]. Table 2 shows the short time adsorption limits for a given interfacial area (technique specific) and the time range that is possible for each dynamic surface tension measurement as reviewed by Eastoe [135] and now including our new MIAM technique. Currently, the Maximum Bubble Pressure Method (MBPM) provides the fastest time scale for the measurement of dynamic surface tension—less than milliseconds [136,137,138].

The other methods, Oscillating jet [143,144], Growing-drop [141,142], Pendant bubble tensiometry (PBT) [139] achieve times down to 10^−3^–10^−1^ s. The Pulsating Bubble Surfactometer is limited to times ~1 s, and the Langmuir Trough/Wilhelmy plate is best used for equilibrium tensions, and only has dynamic times on the order of >20 s because of its large interfacial area ≥10^2^ cm^2^. In comparison, our new Micropipette Interfacial Area-expansion Method technique can make sub-second measurements, but still, there is a three-orders-of-magnitude range difference between the best MBPM and the MIAM [7,8]. However, the slightly slower measurement time for dynamic surface tension for MIAM does not result in a lack of sensitivity. In fact, sensitivity is the other important factor for dynamic adsorption measurements. Since dynamic surface tension measurements monitor the adsorption of the surface active molecular species at the interfacial area, our smaller (microscale) surface and interfacial areas do show higher sensitivity of the dynamic surface tension change at these short times. Compared with the other methods, MIAM, has a meniscus-area in the micropipette of 10^3^−10^5^ µm^2^, (10^−9^–10^−7^ m^2^), which is two to four orders of magnitude smaller area than the other measurement methods (Table 2). Therefore, we can see that MIAM has the highest sensitivity on the list for dynamic surface tension measurements and, consistent with the scope of this special issue, we are making tension measurements at microscales. 

### 4.3. Practical Limits of Theoretical Analysis

For theoretical analysis of the first events in the adsorption of surfactants to a clean surface or interface, in 1946 Ward and Tordai introduced a fairly complex model [151]. It was subsequently developed as asymptotic solutions by Fainerman et al. [152]. A numerical solution by Li et al. [153] describes how the adsorption kinetics of a non-ionic soluble surfactant is governed by a two-step process: (1) the diffusion of molecules from the bulk solution to the subsurface (i.e., the layer immediately below the surface layer, at a thickness of only a few molecular diameters); (2) the molecular diffusion and adsorption from the subsurface layer to the interface [135,154]. It was this model that we used for linearly fitting our experimental dynamic surface tension data for adsorption [7]. The main result was that we could calculate the diffusion coefficient for octanol in this process. Then, using a second micropipette technique, we carried out a droplet dissolution experiment and provide a corroborative measure of octanol diffusion [7]. Therefore, here two micropipette techniques were combined to measure and validate fundamental properties that govern the dynamics of adsorption to surfaces, change of surface tension, and also droplet dissolution. 

For practical purposes, it is now recognised that the Ward Tordai short-time approximation method is valid only over a very specific ranges of time intervals or surface tensions. There is however a “long time” analysis (from ~sub-second to minutes (or hours)) that can give dynamic data. Addressing these issues, MIAM was developed to take advantage of the highest sensitivity for long-time adsorption measurements [7,8]. As described above (Section 2.5. and Figure 10), MIAM is a new tool for the “long-time” adsorption measurements, (that are nevertheless still relatively short at 0.5–1 s) and was used for measuring both equilibrium and dynamic surface tensions for water-soluble surfactants and (later) water-insoluble lipids. 

### 4.4. Dynamic Surface Tension: 1-Octanol and SDS Adsorption

#### 4.4.1. 1-Octanol

**Figure 14:**
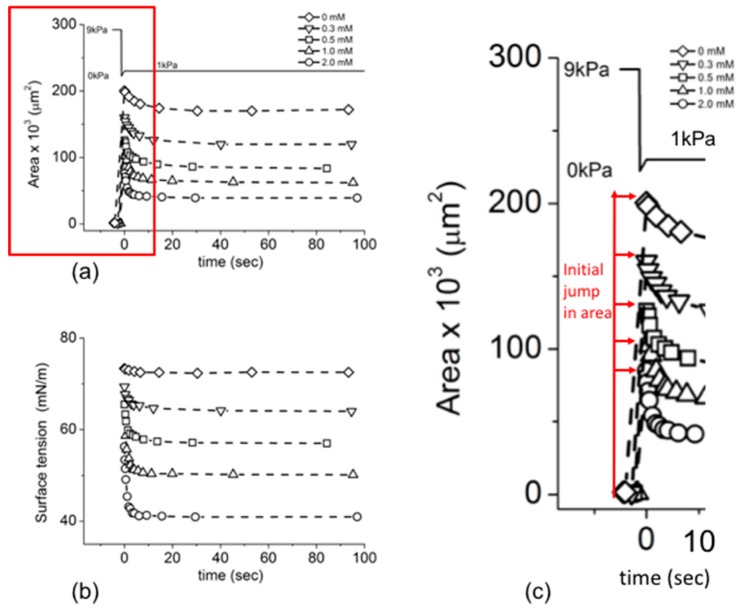
Dynamic surface area and corresponding surface tension of 1-Octanol aqueous solutions at various concentrations measured by the MIAM at 20 °C. (**a**) Meniscus surface-area change vs. time; also shown are the step changes in pipette pressure in sequence, 9, 0 and 1 kPa, and (**b**) derived dynamic surface tension plots also as a function of time. (**c**) Expanded view of the first 10 s for the area vs. time plot, showing more clearly how the meniscus area jumps to smaller and smaller distances, and falls much more precipitously with increasing octanol concentration. With permission from Elsevier [7].

MIAM was used to measure the dynamic surface tension associated with the formation of a 1-Octanol monolayer at the air-water surface [7]. Taking the area changes first, Figure 14a shows the measured area of the small initial air-water surface positioned close to the micropipette tip at a few seconds before time zero (under high 9 kPa applied pipette pressure). This is followed by the rapid area expansion due to the rapid drop in pipette pressure (to 0 kPa), immediately followed by the re-set pressure of 1 kPa (see Figure 10 and Section 2.5 for the protocol). 

For zero octanol concentration, (i.e., pure water), there is a rapid increase in meniscus area from the 1000 μm^2^ at the micropipette tip as it shoots back down the micropipette when the holding high pressure (9 kPa) is taken off to 200,000 μm^2^ further down the taper. As mentioned previously in the protocol, this is a 200-fold area expansion that creates an essentially clean air-water surface in the micropipette. This technique then effectively dilutes any initial concentration of the surfactant adsorbed material at the small surface to ~0.5% and provides essentially a clean surface for subsequent adsorption. Upon application of the new reset pressure of 1 kPa, the meniscus moves to a new position down the pipette, and so there is small decrease in the meniscus area that stabilises within ~5 s. The same measurement was then made for four different 1-Octanol concentrations, 0.3, 0.5, 1.0 and 2.0 mM. Now, in Figure 14a, and expanded in Figure 14c, we see how, with increasing 1-Octanol concentration, the initial jump in area expansion became smaller. As the red arrows indicate, for 0.3 mM it reaches 160,000 μm^2^; for 0.5 mM, 140,000 μm^2^; for 1.0 mM, 110,000 μm^2^ and for 2.0 mM 90,000 μm^2^. This was because of a faster and greater adsorption of 1-Octanol to the surface at higher 1-Octanol concentrations in the short-time of the pressure change. Then, the meniscus area falls much more precipitously with increasing octanol concentration. 

Since this area reduction following the initial rapid expansion was carried out at a constant applied pipette pressure, these meniscus radii were readily converted to surface tensions by applying the Laplace equation to the measured meniscus radii. Analysis of this dynamic area change in terms of a surface tension change is thus shown in Figure 14b for each concentration over the same time period. The pure water control (0 mM, diamonds) showed a slight apparent decrease in surface tension, which would not be expected to occur for a clean air water surface. In the paper we interpreted this as due to a transient heating of the air in the tube connecting the micropipette to the syringe pump, causing slight friction from a rapid plunging of the piston that was nevertheless self-correcting. Then, for the octanol solutions, Figure 14b shows that once the maximum area was attained for this relatively clean surface, the surface tension quickly dropped in a few seconds from ~72 mN/m (pure water surface tension against air, Figure 7c) to succeedingly lower values depending on the degree of 1-Octanol adsorption. It then slowly decreased toward the minimum surface tension, i.e., the equilibrium surface tension, for each concentration. These data then show that octanol rapidly adsorbs to a clean interface and can produce equilibrium tensions within 2–3 s. The data were then analysed by the Ward–Tordai model (as described later in Section 4.5) to obtain the diffusion coefficient for octanol in water.

#### 4.4.2. Sodium Dodecyl Sulfate

**Figure 15:**
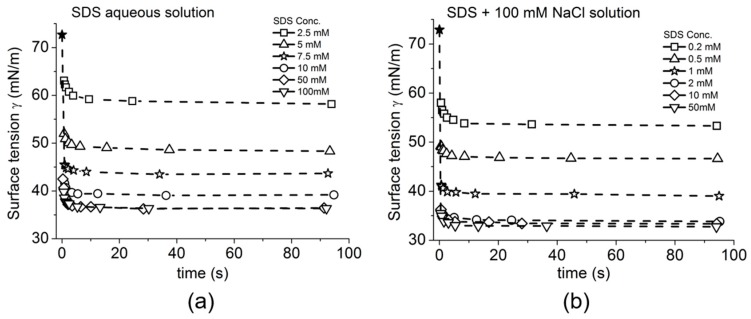
Dynamic surface tension of SDS in aqueous solution and in 100 mM NaCl solution measured by the MIAM at 20 °C. (**a**) SDS adsorption in aqueous solutions for SDS concentrations from 2.5 to 100 mM made up in milli-pure water. (**b**) SDS adsorption in the presence of 100 mM NaCl for SDS concentrations from 0.2 to 50 mM. The solid star symbol (★) shows the initial clean water or 100 mM NaCl surface tensions γ_0_, at time 0 s, obtained from independent measurements. With permission from Elsevier [8].

Similarly, dynamic adsorption of SDS at the air-water surface was also investigated by using the same MIAM technique [8]. As mentioned earlier (Section 3, Figure 11b), SDS is in the form of micelles at and above its CMC (= 8.1 mM in water, and 1.4 mM in 100 mM NaCl). Therefore, a series of dynamic surface tension with different SDS concentrations, 2.5, 5, 7.5, 10, 50 and 100 mM, in water bracketed the CMC. As above described for octanol, based on the area changes for the meniscus, in Figure 15a, where solutions were made up in milli-pure water, we obtained the dynamic surface tension change versus time for each SDS concentration. Figure 15b shows the dynamic surface tension for SDS concentrations of 0.2 to 50 mM made up in 100 mM NaCl.

Just like for 1-Octanol, and as shown in Figure 15a, upon rapid expansion of the initial surface, the dynamic surface tension quickly dropped in a few seconds and slowly approached the equilibrium surface tension for each SDS concentration. Again, this was because of increasing SDS adsorption at the air-water surface toward the equilibrium surface concentration. Initial area jumps were again corelated and the surface tension became measurably less with each increase in SDS concentration. When carried out in NaCl solution, MIAM could also detect the effect of ionic strength. Figure 15b shows the dynamic surface tension change of each SDS concentration mad up in 100 mM NaCl versus time, again decreased in the first few seconds toward the equilibrium. The equilibrium values agreed with the known lowering of the CMC from 8.1 to 1.4 mM by adding NaCl in agreement with Figure 13. The Ward–Tordai analysis then allowed us to estimate the diffusion coefficients of these amphiphilic molecules [7,8].

### 4.5. Ward–Tordai Long-Time Adsorption Analyses for 1-Octanol and SDS Adsorption

**Figure 16:**
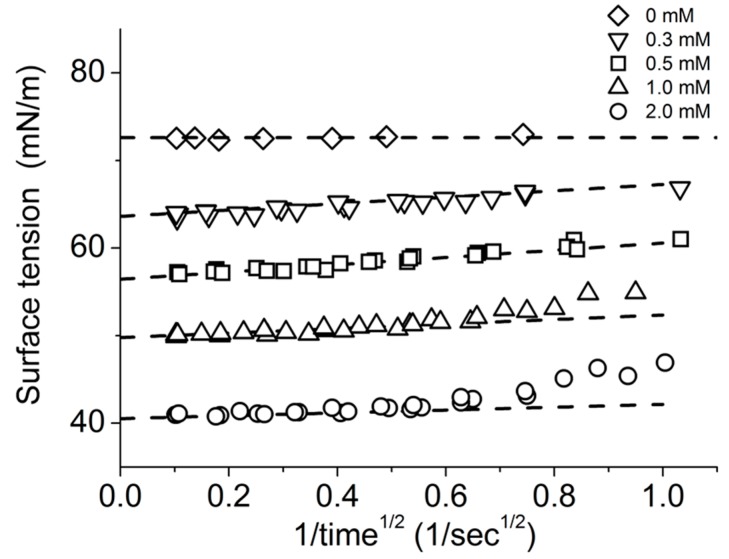
1-Octanol dynamic surface tension at 20 °C plotted according to the Ward–Tordai long-time approximation model Equation (7). 1-Octanol surface tensions from Figure 14b are plotted as a function of *t*^−1/2^. The dashed lines show the best fitting line at each concentration. With permission from Elsevier [7].

As mentioned above (Section 2.5.), the MIAM is designed to measure the surface tension in the relatively “long-time” adsorption regime, albeit with high sensitivity. Therefore, the obtained dynamic surface tension data (Figure 14 and Figure 15) was analysed by using the long-time adsorption approximation of the Ward–Tordai model [7], combined with an adsorption equation to give the surface excess concentration Γ_eq_. Thus, the dynamic surface tension change *γ*(*t*) is given by this Ward–Tordai long-time adsorption approximation as,

(7) $$\gamma \left(t\right)=\gamma_{\mathrm{eq}}+\frac{nRT\Gamma_{\mathrm{eq}}{}^{2}}{c_{0}}\sqrt{\frac{\pi }{4Dt}},$$

where, *n* is a constant, the value of which depends on the amphiphilic compound and salt concentration (if the compound has a charge). In the case of 1-Octanol (non-ionic compound) or univalent ionic surfactant with excess electrolyte with common counterion, *n* = 1; for a univalent ionic compound in the absence of supporting electrolyte, (e.g., in water) it takes on the maximum value, i.e., *n* = 2. *R* is the gas constant, *T* is temperature, *C*_0_ is the bulk concentration. Here, the surface tension is proportional to *t*^−1/2^ by taking a limit of subsurface concentration to bulk concentration in this long-time approximation. Following Equation (7), the surface tension data were replotted as in Figure 16 for 1-Octanol as *γ*(*t*) vs. *t*^−1/2^. 

The data show fairly linear regimes of surface tension versus *t*^−1/2^ for each concentration. Following Equation (7), the linear slope depends on two parameters, i.e., the surface excess concentration at equilibrium, Γ_eq_, and the diffusion coefficient, *D*, of the compound in aqueous solution. Therefore, to estimate the D value from the slope of dynamic surface tension change, we needed to determine the Γ_eq_ value at each concentration of the surface active compound. Unfortunately, the Γ_eq_ value is not obtainable for our measurements. Although the Γ_eq_ value is measurable directly by using other techniques, e.g., neutron reflection, the technique needs further development for direct dynamic Γ(*t*) measurements [155,156,157]. Alternatively, we can estimate the maximum surface excess concentration, Γ_m_, value from fitting models, such as Langmuir isotherm and Frumkin isotherm models, to equilibrium surface tension plotted against the logarithm of concentration. The maximum surface excess concentration, Γ_m_, values were in turn obtained from fitting adsorption isotherm models to equilibrium surface tension plotted against the logarithm of concentration. We found that the best isotherm was the extended Frumkin isotherm adsorption model with a deal ionic activity correction factor. The estimated Γ_m_ (= 7.75 × 10^−6^ mol/m^2^) from MIAM [7] was in the same range as other methods, ~6–9 × 10^−6^ mol/m^2^ [139,143,150,158], giving diffusion coefficient values from the slopes in Figure 16 of 7.2 ± 0.8 × 10^−6^ cm^2^/s for the Frumkin model. Interestingly, using a different micropipette technique we could also check this value by carrying out a droplet dissolution experiment for single 1-Octanol microdroplets that we reported in the same paper [7]. The diffusion coefficient D value of 1-Octanol was also directly measured by droplet dissolution and analysed by the Epstein-Plesset model giving *D* = 7.3 ± 0.1 × 10^−6^ cm^2^/s, which showed excellent agreement between these dynamic surface tension data. Thus, for non-ionic surface active compounds, the adsorption barrier and activation energy derived from the adsorption dynamic models (especially the Frumkin model) worked well to explain the obtained dynamic surface tension data and also the diffusion coefficient [7,139]. Both obtained D values showed excellent agreement compared with other dynamic surface tension measurement methods, such as pendant bubble method [139].

**Figure 17:**
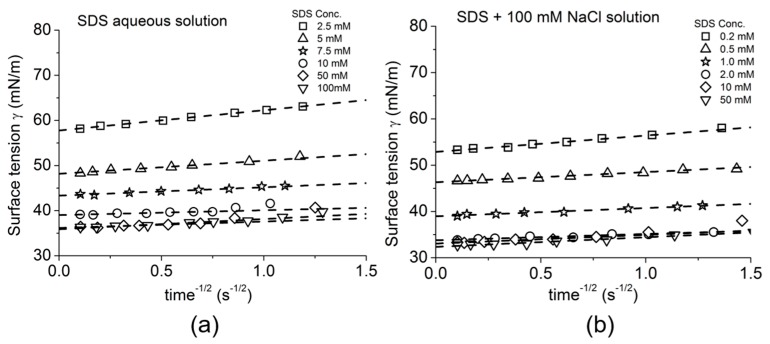
SDS dynamic surface tension at 20 °C plotted according to the Ward–Tordai long-time approximation model Equation (7). SDS surface tensions are plotted as a function of *t*^−1/2^. (**a**) SDS surface tensions for different SDS aqueous solution concentrations made up in milli pure water from 2.5 to 100 mM. (**b**) SDS surface tensions for different SDS solution concentrations from 0.2 to 50 mM, made up in 100 mM NaCl. The intercept of each fitting line showed agreement with the equilibrium surface tension at each concentration. With permission from Elsevier [8].

We also applied the long-time adsorption approximation model to the dynamic surface tension for SDS [8]. Again, the dynamic tension data was replotted with the time axis of *t*^−1/2^. Figure 17 shows the replotted results of SDS in the absence and presence of salt.

In both cases, whether in the absence (Figure 17a) or presence (Figure 17b) of salt, the obtained dynamic surface tension data followed a linear slope for each concentration. Analysis for this case of an ionic surfactant was much more complicated than the non-ionic octanol and required corrections to several parameters used in the analyses. First, as described in more detail in the paper [8], the maximum surface excess concentration, Γ_m_, according to the Frumkin isotherm did not account for the adsorption activation energy ΔE of ionic surfactant. This inconsistency was solved by applying a mean ionic activity correction to the fitting [159]. Then, an ideal ionic activity correction factor A_±i_ (= 0.29) for SDS was required and was obtained from the plot of mean ionic activity coefficient vs. Γ_m_ measured at different salt concentrations. This treatment of A_±i_ provided a means to normalise to a condition representative of a non-ionic interaction for an ideal electrolyte solution of SDS, i.e., the SDS molecules would be able to diffuse and adsorb at the air-water surface just like a non-ionic surfactant in this condition [8]. After applying the A_±i_ correcting factor, the mean ionic activity became the “ideal” ionic activity, A_±i_C*, and gave a more consistent *D* value, *D* = 5.3 ± 0.3 × 10^−6^ cm^2^/s for the diffusion coefficient of single SDS molecules in aqueous media. 

## 5. Equilibrium and Dynamic Surface Tension: Adsorption of Insoluble Surfactants

In this final fundamental section, we review the measurement of equilibrium and dynamic surface tensions that we have made using the micropipette technique for insoluble lipid monolayers. Here, again using the tapered micropipette manipulation techniques [6,9], we chose to study a homologous series of phospholipids as our test materials. Later, in Section 6 Applications, we also include an interesting application that was initiated based on these earlier experiments—measuring the equilibrium and dynamic surface tensions and observing the kinds of multi-layered structures that occur at the air-water surface for natural and synthetic lung surfactants composed of mixed lipid and protein/peptide surfactant [10].

### 5.1. Equilibrium Surface Tension for Adsorption of Phospholipids vs. Temperature

**Figure 18:**
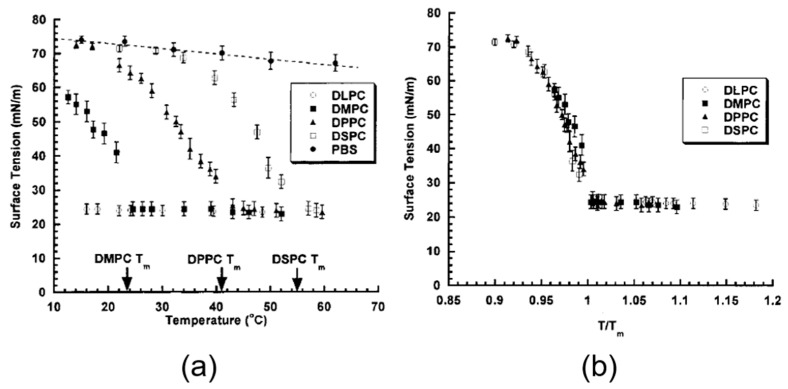
Equilibrium surface tension of four different phospholipids, i.e., dilauroyl-phosphatidylcholine (DLPC), dimyristoyl-phosphatidylcholine (DMPC), dipalmitoyl-phosphatidylcholine (DPPC) and distearoyl-phosphatidylcholine (DSPC) monolayers. (**a**) Equilibrium surface tension of each phospholipid monolayer at each temperature. Arrows mark the gel-to-liquid crystalline phase transition temperature for each phospholipid. **(b**) The equilibrium surface tension values are plotted as a function of their relative phase transition temperature (T/T_m_), and the behaviour is collapsed to a single curve. Reproduced from Lee et al. [6], with permission from American Chemical Society.

In the early work by Lee et al. [6], we used that original tapered micropipette manipulation technique to study the homologous series of phosphatidylcholines, increasing chain length (e.g., diC12–diC18) produces an increase in their main acyl chain melting transition temperatures (T_m_) [160]. Figure 18 shows the equilibrium surface tension of four different saturated phospholipids, dilauroyl-phosphatidylcholine (DLPC, C12:0), dimyristoyl-phosphatidylcholine (DMPC, C14:0), dipalmitoyl-phosphatidylcholine (DPPC, C16:0), and distearoyl-phosphatidylcholine (DSPC, C18:0) in phosphate buffer (pH 7.4), PBS, with increasing temperature [6]. 

As a control, it is important to recognise that the clean PBS-interface has a slight temperature dependence, decreasing from 74 mN/m at 13 °C to 66 mN/m at 62 °C, but this in no way can account for the decreases seen in the presence of the phospholipids. Starting at low temperatures relative to their respective gel-to-liquid crystalline phase transition temperature *T_m_*, the measured equilibrium surface tension (Figure 18a) of each phospholipid progressively decreased with increasing temperature. The surface tensions for all phospholipids reached a lower minimum constant value of ~24 mN/m, coinciding with each *T_m_* (23.5, 41.4 and 55.1 °C for DMPC, DPPC, and DSPC, respectively). Above this temperature, the surface tensions were relatively constant for these now liquid phase monolayers.

This constant value above *T_m_* suggests there is no influence of carbon chain length between C12 and C18 on the *γ_m_* value for the liquid crystalline phase. Moreover, when the same plots were replotted scaling the temperature as a function of their relative phase transition temperature (*T/T_m_*), Figure 18b showed that all the data from all four different lipids collapsed onto a single curve. Thus, the temperature-dependent change in surface tension (decreasing with increasing temperature, until a minimum value is reached) only depends on the relative phase transition temperature, and not on the total carbon chain length. What this data then shows is that the limiting surface tension is governed by the hydrocarbon-air interface of the outer parts of the chain and terminal methyls. In fact, the common value of *γ_m_* ~ 24 mN/m is essentially the same as that measured for liquid hydrocarbons like n-decane which has a decane-air surface tension of 23.7 mN/m at *T* = 22 °C [161]. 

### 5.2. Dynamic Surface Tension for Adsorption of Phospholipids

**Figure 19:**
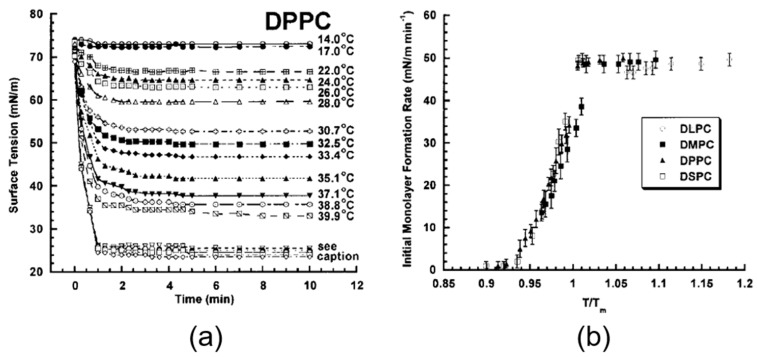
Dynamic surface tension change and initial monolayer formation rates. (**a**) Effect of temperature on adsorption kinetics at the water-air interface of DPPC monolayers, as monitored by change in surface tension with elapsed time of exposure of the interface to DPPC lipid aqueous suspensions (1.0 mM) at various temperatures from 14 to 59.6 °C. Note: the surface tensions at (43 °C (∇), 45 °C (●), 47 °C (○), 51.1 °C (□), and 59.6 °C (◊) all rapidly reached an equilibrium value of 25 mN/m within 1 to 2 min, and so the data effectively lies on top of each other. (**b**) Plot of the initial monolayer formation rates of four different lipids, i.e., DLPC, DMPC, DPPC, and DSPC, at the air-water surface as a function of their relative phase transition temperature (*T/T_m_*). The four different types of lipid data were collapsed to a single curve. Reproduced from Lee et al. [6], with permission from American Chemical Society.

We also made dynamic surface tension measurements for these same insoluble lipid monolayers. Here, only the simple area-expansion method was used and so we only observed the interface after about 15 s of initial monolayer formation [6]. However, this did not pose too much of a problem in time resolution because, unlike the molecular species of octanol and SDS, the adsorbing species were liposomes, and so the rate of monolayer formation reflected the diffusion (Brownian motion) of these much larger, 100 nm entities. Diffusion coefficients for liposomes (250 nm) have been measured in water to be ~8.8 × 10^−8^ cm^2^/s [162], and so liposome diffusion is over 100 times slower than simple single molecules (7.3 and 2–6 × 10^−6^ cm^2^/s for octanol and SDS respectively). Such entities could be subject to Ward–Tordai analyses (but have not been done so far). In any event, the process of adsorption and monolayer formation from liposomes is not just governed solely by their diffusion to the interface; it also includes their collapse and spreading, and so there are potentially multiple rate determining steps. In this experiment then, we could still observe the monolayer formation rate as liposomes spread and lowered the interfacial tension. Figure 19a shows the dynamic surface tension change for just one of the lipids, DPPC, with increasing temperature. 

DPPC has a main acyl meting temperature *T_m_* of 41.3 °C when it enters the liquid Lα phase. Below this temperature, the liposomes are in their so called “gel” phase, where, for DPPC, there are also several sub-phase transitions with distinct temperature ranges [160]:

- P_β_’ or ripple phase from 34.45–41.3 °C
- L_β_ planar gel phase from 18–34.4 °C

Therefore, the temperature ranges in Figure 19 were designed to capture these phases up to and including the melted liquid L_α_ phase.

As is shown by the Figure 19a, at 14 °C, which is below its L_c_ phase, DPPC liposomes do not hardly, if at all, even spread on the clean 72 mN/m air-water surface. With successively increasing temperatures, the equilibrium surface tension is lowered at faster and faster rates through each bilayer sub-gel-phase until the liquid state is reached and the minimum surface tension is obtained. As concluded by Lee et al., in order for rapid spreading on the clean interface to occur the lipid vesicles are required to be in the liquid state. Additionally, the equilibrium surface tension and the monolayer formation rate depend on the relative phase transition temperature of the lipid. Thus, we see that, in agreement with MacDonald and Simon’s study on a similar lipid DMPC [117], the monolayers follow the bilayer systems (liposomes) with which they are in equilibrium.

As with the surface tension data vs. reduced temperature (T/Tm), the monolayer formation rate (in units of mN/m·min), shown in Figure 19b, also collapsed onto a single curve for all lipid systems. Interestingly, at a reduced temperature of 0.9 to 0.93 for all lipid systems, none of the lipids would spread on a clean interface. Then, from 0.93 to 1.0 there was an increase in the spreading rate until T_m_, at which point the initial monolayer formation rate reached a common maximum of ~50 mN/m·min. Comparisons with previously reported data using the Langmuir trough can be made with this micropipette-lipid-adsorption data. The Langmuir trough produces surface pressure isotherms, and so we can convert the surface tension values of the lipid-adsorbed interface (*γ*) and the clean interface (*γ_o_*) into surface pressure values (*π*) via the relation *π* = *γ_o_* − *γ*. Converting the data in Figure 18b, to surface pressure (surface pressure is negative) a nearly linear increase in equilibrium spreading pressure for the monolayer material was observed as the temperature was raised to the transition temperature.

Thus, using the tapered micropipette manipulation techniques, Lee et al. (2001) found that both equilibrium and dynamic surface tensions depend on the relative phase transition temperature T/T_m_ of the lipid [6]. One interesting observation here is that for all lipid systems, for a reduced temperature of 0.9 to 0.93, lipids actually have zero spreading pressure on a clean interface. One could imagine a practical application of this result where such highly solidified lipid vesicles could be used in aqueous suspension to actually keep interfaces clean by perhaps acting as sinks for other impurity materials.

## 6. Applications in Medical Imaging (Ultrasound), Oil Recovery, Nanoprecipitation, the Biology of the Lung Interface, and Microfluidics

Finally, we give examples of where our measurements of micro-surface tensions, and those of others (notable Tony Yeung [11]), for clean and adsorbed surfaces, and gas- and droplet-dissolution have had an impact on five applications. These selected applications include: (1) gas microbubbles for ultrasound contrast; (2) interfacial tensions for micro-oil droplets in oil recovery; (3) surface tensions and tensions-in-the surface for natural and synthetic lung surfactants; (4) interfacial tension in nanoprecipitation; and (5) micro-surface tensions and droplet dissolution in microfluidics.

### 6.1. Gas Micro-Bubbles for Ultrasound Contrast: Surface Tension and their Dissolution into Water at the Scale of the Microbubble

Measuring the surface tension of an air-water surface [6,9] and then being motivated by industrial interest to working with micro bubbles [12] was actually the first time we developed the micropipette technique from its original applications in studying cells and vesicles to studying colloids and surfaces of gas, liquid and solid microsystems. As we saw in Figure 5 and Figure 7, just by placing an air-filled pipette in a water filled chamber it is simply a matter of increasing the positive pipette pressure to drive the gas interface to the tip of the pipette. Applying a bit more pressure then blows out a gas microbubble bubble. The additional pressure required to do this is relatively small compared to the large pressure required to get the meniscus to such a small radius of curvature. As described by Tony Yeung [11] (see Section 6.2. where we give the equation for aspirating a droplet, but now consider the process is in reverse), when an air-water surface inside the pipette is driven by positive pressure to emerge from the pipette tip into the unconstrained environment of the chamber, the formation of a bubble of diameter greater than the pipette tip diameter results in the rapid growth of the gas bubble. As can now be appreciated, this pressure is determined by the level of surface tension of the surface itself, and, if it is a clean air-water surface of 72 mN/m, the pressure needed to blow out the bubble through the relatively small micropipette tip, becomes a large driving force for microbubble expansion. The force balance associated with the buoyancy of air will pull the forming bubble off the pipette at some point, but microbubbles can grow to hundreds of micrometres before they detach. One technique we developed to control the formed microbubble size was to simply angle the pipette at the top of the chamber so that its tip was 10–20 μm below the glass surface such that as the microbubble rapidly emerged, it was forced off the pipette. We could therefore “manufacture” a series of gas microbubbles by this angled-pipette technique and then pick single microbubbles for study.

In this application the goal was to stabilise gas microbubbles against dissolution for an important medical application—ultrasound contrast. While gassed-up salt solution had been an industry standard, at the time new contrast agents were being formed using proteins [163]. One was called Albunex (Molecular Biosystems, Inc, San Diego). It was a commercially prepared contrast agent made from sonicated 5% human serum albumin. The mean microsphere size used in these experiments was 4.0 μm with a concentration of 437 million/mL. The (competing) company who contracted us, was interested in, if, and to what extent, lipids could be used as the stabilising monolayer. As with Albunex, the idea was that following intravenous injection, they could survive long enough in the blood stream to provide blood-pool contrast using ultrasound for myocardial contrast echocardiography (MCE) and the like. As was obvious from Laplace surfaces, surface tension is a key parameter that determines the dissolution rate of air into water because the surface tension creates an above-ambient pressure on the gas inside the microbubble, as given by the Laplace equation, Equation (5). The influence of surface tension and the role of microbubble size, and the diffusion coefficient of air in water were the key parameters that Epstein and Plesset [96] brought together in their 1950 paper that described gas bubble dissolution (or growth). Therefore, we started our product development by testing and validating this equation using gas microbubbles made and observed using the micropipette technique. As we will see, this is again where, working at the microscale meant we could observe and measure all dissolution processes for single individual microbubbles in a convenient lab time frame of just a few seconds.

The basic Epstein–Plesset (EP) equation for the dissolution rate of a free gas microparticle considering only the gas concentration in an unsaturated solution (surface tension is not yet included) is given by,

(8) $$\frac{dR}{dt}=-\frac{D\left(C_{s}-C_{o}\right)}{\rho }\;\left[\frac{1}{R}+\frac{1}{\sqrt{\pi Dt}}\right],$$

where *R* is the diameter of the microbubble, *C_s_* is the saturation concentration, *C_o_* is the concentration of gas in the bulk solution, *ρ* is the density of the gas, and *t* is time. The model assumes that there is a large volume of surrounding solution relative to the volume of the bubble, the gas concentration at the bubble’s surface is in equilibrium with the gas in the bubble and is considered saturated, *C_s_*, and the concentration at infinity is the initial gas concentration in solution *C_o_*.

The inclusion of surface tension enters the analysis through of the curvature of the bubble’s surface and the fact that a surface tension at the interface creates an over pressure inside the bubble according to the Laplace equation, (given earlier, Equation (5)). The total pressure in the bubble therefore increases as *R* decreases. Thus, the influence of surface tension is introduced into the dynamic equation, assuming ideal gas, via a recalculation of the gas density with increasing over-pressure as given by Epstein and Plesset [96] and detailed in Duncan and Needham [12].

Until we did the experiment over 50 years later using the micropipette technique [12], this equation, and the dissolution rate as influenced by the surface tension, had never really been tested at the scale of gas microbubbles. As already mentioned, it was the ability to work at this scale of 10 s of microns using the micropipette technique, that allowed the gas bubble size and dissolution rate to be measured, whereas all previous attempts using millimetre sized bubbles took days to dissolve, making it difficult to readily test this important model for gas-dissolution. We have also now extended these studies and this model fully characterises the dissolution of immiscible liquid microdroplets of oil into water and water into oil, [95,164,165].

#### 6.1.1. Test of the Epstein–Plesset Model for Gas Microparticle Dissolution in Aqueous Media

**Figure 20:**
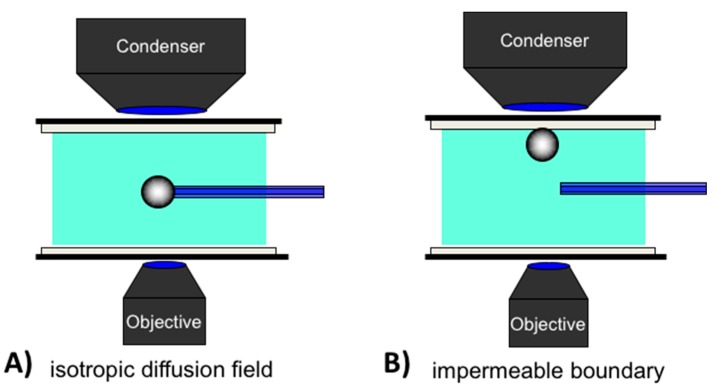
Schematics of pipette and gas microbubble in microchamber. (**A**) Microbubble held in infinite dilution and isotropic diffusion field; (**B**) microbubble allowed to rise to the top of the chamber, also in infinite dilution but now air-diffusion is limited by the impermeable boundary of the wall of the glass microchamber.

The experiment then was to simply form a 10–20 μm diameter air microbubble using the micropipette, and, as shown in Figure 20A, hold it on the tip of the pipette, in a static position, in infinite dilution, in an isotropic diffusion field, in the middle of the aqueous filled chamber. 

As shown in Figure 20A, we could then observe its gradual dissolution into saturated or undersaturated water, and fit this data to the EP model knowing the surface tension of the air-water surface. Air bubbles were better stabilised (against adhesion to the glass pipette) by the adsorption of an SDS monolayer and so were formed in a 10 mM SDS solution giving an air-water SDS monolayer surface tension of 40 mN/m. In Figure 20B, the microbubble is released from the micropipette and allowed to rise to the top surface of the chamber. There is now an impermeable boundary that limits the air diffusion away from the microbubble into the aqueous phase, and so the microbubble is no longer in an isotropic diffusion field, but this can be accounted for by an empirical model [12,166]. 

**Figure 21:**
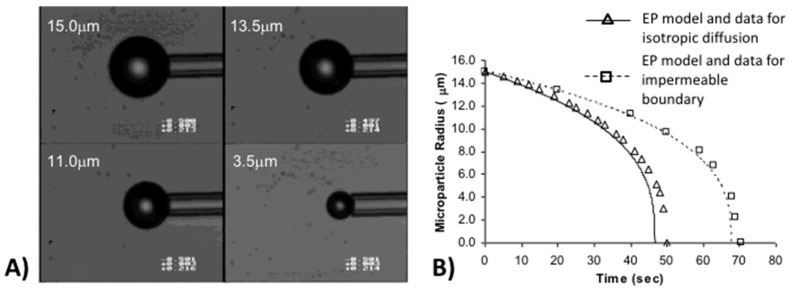
Dissolution of a 30 μm diameter air bubble into water. (**A**) A series of video images of the dissolution of an air microbubble in 10 mM SDS solution held in the center of the chamber by a pipet with a very low suction pressure of 500 Pa and 21.5 °C. The air bubble is shown at times 0 s (top left), 10 s (top right), 30 s (bottom left), and 48 s (bottom right) respectively, completely dissolving in ~50 s. (**B**) Plot of microparticle radius versus time for dissolution in isotropic and boundary-limited conditions where the data are fitted by the Epstein–Plesset (EP) model and an empirical model by Wise et al. [166].

Figure 21 shows the data along with the EP model with no free parameters, thereby validating this model for an air-water surface stabilised by the soluble surfactant SDS. Thus, a 30 μm diameter air microbubble dissolves in water in 50 s, in almost complete agreement with the EP model. Imagine now how compromised the original contrast agent systems were of gassed up salt solution! How long would a 5 μm diameter gas bubble would last in the blood stream? Just a few seconds! Of course, if it adsorbed any protein, that would reduce its surface tension (see next Section 6.1.2.), then it might last a little longer. However, initially-uncoated gas microparticles were extremely difficult to work with and not very effective contrast agents. Now we know why, quantitatively.

A released microbubble though (Figure 20B) rises to the top of the chamber and comes to rest against the glass surface. The result is that air saturation of the solution can build up around the microbubble and so its dissolution is ~44% longer. The data is fit to an empirical model by Wise et al., [166] in Figure 21B. We mention this boundary condition here because it may be of interest and important to the current “Micromachine” audience since microbubbles near surfaces or in bulk-isotropy occur in one or more of the in micro- and nano-systems (that might include, microfluidic and lab-on-chip devices, soft gripping and manipulation of particles, colloidal and interfacial assemblies, fluidic/droplet mechatronics).

Gas microbubble-lifetimes depend on both the dissolution-driving over pressure due to their surface tension and the diffusion field that surrounds the microbubble. This boundary condition will also apply to dissolving micro and nano-scale liquid-in-liquid emulsions, but here the over pressure is negligible because of the incompressibility of liquids as we have also shown for an aniline-water micro-system [164]. Additionally, as is clear from the EP model, an increase in surface tension results in a faster dissolution and so shorter dissolution time. For example, the same 30 μm diameter air bubble for a clean air-water surface tension of 72 mN/m would take ~30 s to dissolve compared to 50 s for the lower surface tension (40 mN/m) of the SDS-coated bubble.

#### 6.1.2. When the Tension-in-the-Surface is Zero: Effect of Gas Saturation in Solution on Microbubble Dissolution

Since the surface tension creates the over pressure, gas bubbles will always dissolve even in saturated solution. However, what would happen without this driving force? That is, what if there was no surface tension at the gas bubble surface? Clearly, for an air-water surface or any interface to exist we cannot have a “zero surface tension.” However, we can have “zero-tension-in-the-surface” if that surface is now a monolayer of an insoluble material, like, for example, a phospholipid. Basically we “coated-air” and, in the same paper [12], we measured the rates of dissolution, in the absence of the Laplace overpressure, for increasing gas saturation in the aqueous medium. In order to create a “zero-tension-in-the-surface” we utilised the same gel-phase DSPC lipid as a monolayer shell adsorbed on the air microparticle, as was used and shown to adsorb by Lee et al. [6] (and described earlier in Figure 18 and Figure 19). This adsorbed, solid-phase, lipid monolayer permits the assumption to be satisfied of zero tension in the gas microparticle surface, and with it, a condition of zero Laplace pressure. Gas dissolution is then only driven by the level of gas under-saturation in the surrounding aqueous phase placed in the microchamber. (Note this is now not a gas-bubble per se with an exchangeable interface composed of a soluble surfactant or just water; it is gas encapsulated in a monomolecular insoluble shell).

**Figure 22:**
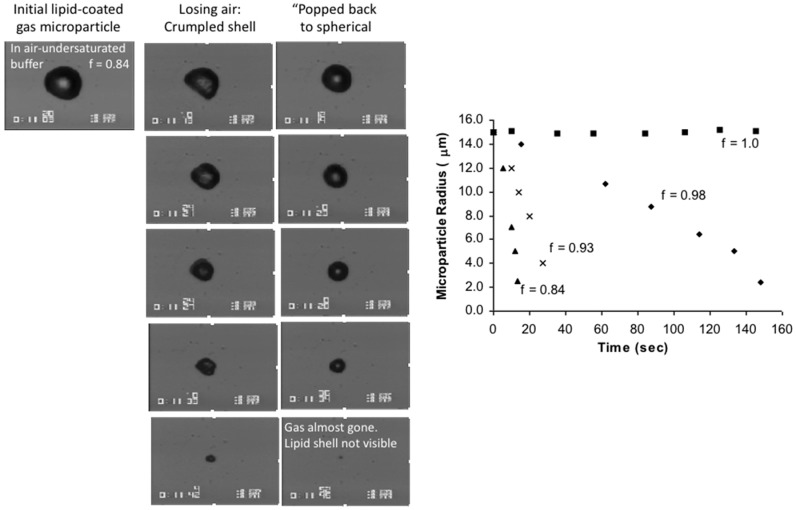
Lipid-coated gas microparticle losing air in undersaturated solution. The degree of air-water saturation, f, is the ratio of the initial air concentration in surrounding water to the saturated concentration of air in water. (**Left**) Videomicrograph of the initial lipid-coated gas microparticle in f = 0.84. (**Middle**) Series of lipid-coated gas-microparticles losing air, the shell crumples, and then pops back to spherical as lipid is shed until there is no gas left; (**Right**) plot of microparticle radius vs. time for gas microparticles losing air at increasing air saturation. The microparticle in the saturated solution (f = 1) is stable.

Starting with a maximally undersaturated solution, and carrying out dissolution-experiments much faster than the undersaturated solution re-saturates with air, we could see that as the air escaped from the gas microparticle (Figure 22), the solid-shelled monolayer was observed to be misshapen (crinkle) and, at excess crumpling, reshaped (“popped”) back to spherical caused by a shedding of the lipid monolayer while still remaining contiguous with the surface material. Under this microscopic resolution the shell is not visible, but was seen with interference optics. This return to a spherical shape allowed us to quantify the gas-loss as a change in radius, measured at each spherical-reshaping, versus time as a function of the initial degree of air-water saturation, f. We confirmed that the particle dissolved at slower and slower rates the closer the degree of under-saturation, f, got to unity. At f = 1, conditions essentially represented an infinite dissolution time, demonstrating that the solid shell indeed provided *zero tension* in the surface and zero Laplace pressure.

While beyond the scope of this paper to go into too much detail, it is worth mentioning briefly that, having formed these “zero-tension” shells, the micropipette technique was also used to characterise the mechanical (viscoelastic) nature of lipid shells on gas microbubble surfaces. Interested readers can learn of these experiments and results in a series of experiments on lipid-coated single gas microparticles [13,14], including the process of making the lipid-coated microparticles in which air micro-bubbles were formed by sonication in the presence of lipid as liposomes above their phase transition temperature. Following the data in Figure 18 and Figure 19, upon formation of the clean gas microbubble air-water surface, lipid rapidly adsorbed (to equilibrium in 2 min) to form monolayer above T_m_, and the final coated microbubble suspension was obtained when it was cooled to solidify the monolayers. Aspiration of the shells demonstrated that they deformed in shear at room temperature and that the values of their yield shear and shear viscosity were dependent on the composition, grain microstructure, and thermal processing of the material [13,14]. For example, the 2D viscosity for the series of diC18–diC24 phosphatidylcholines was dependent on their relative transition temperature, characterising such surface monolayers with values of yield shear (1–6 mN·m^−1^) and shear viscosity (5–25 mN·s·m^−1^) obtained by the micromanipulation technique for these phospholipid shells. These values of yield shear and shear viscosity can be converted to comparative bulk values by dividing by the monolayer thickness of 3 nm, giving values of 1.7–8.3 × 10^6^ Ns/m^2^, (1.7–8.3 MPa) and 0.3–2 × 10^6^ N/m^2^, (0.3–2 MPa·s) which interestingly are comparable to the properties of common plastics like high density polyethylene [167]—tensile yield (10 Mpa) and melt-viscosity (0.3 MPa·s). This again demonstrates that the intermolecular interactions (largely van der Waals-bonding) between lipid acyl chains dominate micromechanical and surface-properties of such monomolecular materials.

### 6.2. Surface Tension Measurements of Microdroplets for Oil Recovery

**Figure 23:**
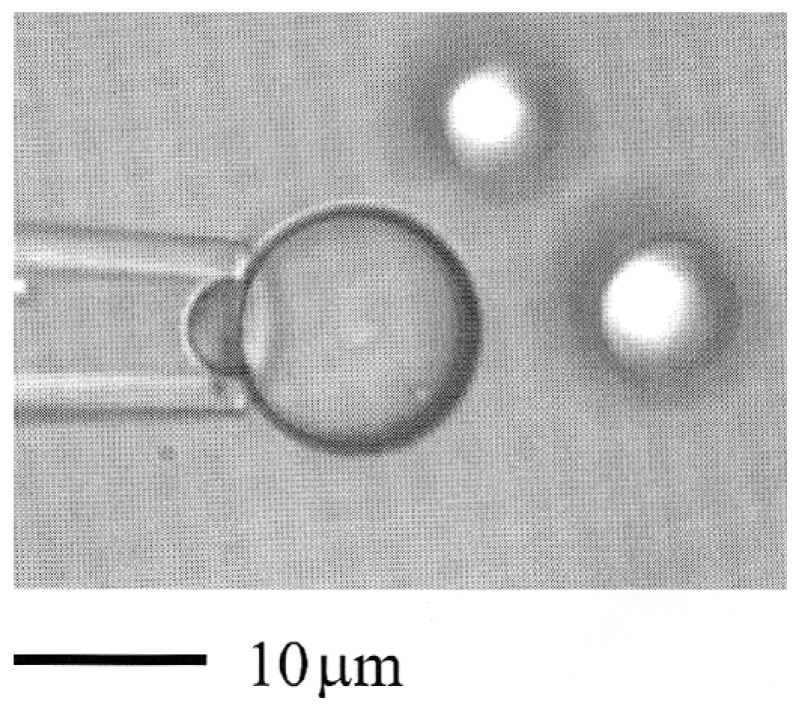
Single water microdroplet held by the micropipette in heptol solution (1:1 mixture, by volume, of *n*-heptane and toluene). The water-oil interfacial tension of 40.3 ± 0.6 mN/m was directly measured from the geometrical shape of the microdroplet and the minimum pressure required to draw in the droplet to give a projection length inside the pipette of one pipette radius. With permission from Elsevier [11].

Instead of making an interface inside the tapered micropipette, we have shown that an isolated single oil droplet can be formed in water [16,17,18,95], or water microdroplet can be formed in oil [165,168] and held at the tip of the micropipette thereby creating a micro-interface. Then, by applying a controlled suction pressure to the oil droplet, it is possible to expand the interface and measure the critical tensile yield (interfacial tension) of the immiscible interface. This unique droplet interfacial tension measurement was used by Yeung’s group in 2000 [11]. Figure 23 shows a single water droplet in heptol (1:1 mixture, by volume, of *n*-heptane and toluene). 

For this water-microdroplet-aspiration technique, the interfacial tension (symbolised now in Yeung’s equations by *σ*), of 40.3 ± 0.6 mN/m was calculated from the geometrical shape of the deformed microdroplet with critical pressure, *p_cr_*, following the relation [11,169],

(9) $$\sigma =\frac{p_{cr}R_{p}}{2\left(1-\frac{R_{p}}{R_{o}}\right)},$$

where *R_p_* is the inner radius of the pipette tip, and *R_o_* is the radius of the exterior drop segment. The equation was originally developed by Evans to measure the lipid bilayer membrane tension in mechanical characterisations of the red blood cell membrane [56,58,63,170] and was also extensively applied to characterise the mechanochemistry of synthetic of phospholipid membranes as giant unilamellar vesicles [73,75,76,77,78,79,80,83,108,118,169,171]. It is thus a main-stay of the micropipette technique. In order to calibrate and validate the droplet-tension technique, interfacial tensions were measured by Yeung et al. for water vs. ethyl acetate, dichloromethane, toluene, carbon tetrachloride and showed fairly good agreements with the literature values. In Yeung’s experience, some oils such as crude oil showed high viscosity and were sticking against the glass pipette walls. To avoid measurement errors, the micropipette surface was salinized [11]. We have also used such salinization when dealing with lung surfactants [10] and these experiments on lung surfactant properties are given next. 

### 6.3. Surface Tension or “Tension-in-the Surface”: The Biology of the Lung-Air Surface

Taking the basic and fundamental measurements of lipid monolayer surface tensions we made in 2001 [6] into an application, we recently carried out a series of interesting and illuminating new studies on natural and synthetic lung surfactants [10]. Surfactant formulations are used for the medical treatment of a range of lung conditions including, neonatal respiratory distress syndrome (NRDS), Acute Lung Injury (ALI) and Acute Respiratory Distress Syndrome (ARDS) patients [172,173,174]. The new micropipette studies show not only the level of surface tension achieved by these components of lipid and protein at the air-water surface, but have also allowed us to visually observe and measure the assembly of new multi-lamellar structures—all by viewing the material adsorption in the tapered-micropipette. Again, with an average diameter of 100–200 μm, our micropipette techniques provide surface tension and structural data at the same scale as the phenomenon under study. In this case adsorption and spreading on alveolar surfaces, that have, themselves, an average diameter of 200 µm, with an increase in diameter and hence expansion in surface area during inhalation—all the parameters (radius of curvature, surface area and applied pressure across and tension in the surface) we can control and apply in the micropipette manipulation techniques. 

#### 6.3.1. Lung Surfactant Components

The major components of natural lung surfactant are lipids (~90% by mass: phosphatidylcholines, phosphatidylglycerol, and cholesterol) and three proteins (~10%: hydrophilic SP-A and SP-D proteins, and hydrophobic SP-B and SP-C proteins) [147,175]. Of these proteins, SP-B (79 amino acids; monomer MW of 8.7 kDa), is strictly required for the assembly of pulmonary surfactant and the formation of stable surface-active films at the air-liquid alveolar interface, making SPB essential for lung expansion function [176]. Nowhere is this more critical than in NRDS in premature babies, [177] who are born without lung surfactant; an absence of lung surfactant is incompatible with life itself.

Currently, there are several commercialised lung surfactant-products available on the market. These include: the animal lung-derived surfactant products, Curosurf, Survanta, Infasurf, Alveofact, and BLES; and synthetically developed products like Lucinactant (Surfaxin), (consisting of phospholipids, a fatty acid, and sinapultide (—a 21-amino acid hydrophobic synthetic peptide called KL4 peptide), and Colfosceril (exosurf) (consisting of just the synthetic lipid Colfosceril Palmitate (DPPC), plus—Cetyl Alcohol, and Tyloxapol—a non-ionic liquid polymer of the alkyl aryl polyether alcohol. In addition to these more traditional animal and synthetic surfactant formulations, Molecular Express and their academic collaborators (Walther and Waring) in California have developed a new, completely synthetic, formulation that mimics the Surfactant Protein (SP)-B, called Mini-B [178], that was recently modified further into a Super Mini-B construct [179]. The Super Mini-B (SMB) analogue, together with a second peptide construct SM-C [180] showed that this combination was actually superior to single-peptide formulations in rabbits with chemical acute lung injury.

Thus, as introduced by Walther et al. [179], Mini-B, (34 amino acid sequence) is a disulphide-linked construct based on the N- and C-terminal regions of SP-B (i.e., residues 8–25 and 63–78). Mini-B retains critical in vitro and in vivo surfactant functions of the native protein, and the Super Mini-B construct has native SP-B residues (1–7) attached to the N-terminus of Mini-B. These peptides are known to rearrange lipid molecules in the fluid lining the lung so that alveoli can more easily inflate. The new synthetic lung surfactant formulation then comprises a host phospholipid mixture (PL = DPPC:POPC:POPG 50:30:20 molar ratio) and the 4 wt% SMB lung surfactant protein peptide. In keeping with the personalised-introduction, the lung surfactant projects started when we were asked by our friend, collaborator and CEO/President of Molecular Express Inc, CA, Gary Fujji, *“Can you measure surface tensions?”* … *“Of course we can, see our papers from 2001”* … and this launched another industrial project. While other techniques, such as the pulsating bubble surfactometer had been used in the past to measure lung surfactant tensions [122], we applied the tapered micropipette manipulation techniques to investigate lung surfactant air-water surface tensions and the multi-layered and complex structures they produced [10]. The hypothesis was that the inclusion of the SMB peptide would induce lipid fusion and massive reassembly of multilamellar structures, and that we could observed this at the microscopic interface in the micropipette. This was indeed the case.

#### 6.3.2. Equilibrium and Dynamic Surface Tensions for the Lung Surfactant Formulations

**Figure 24:**
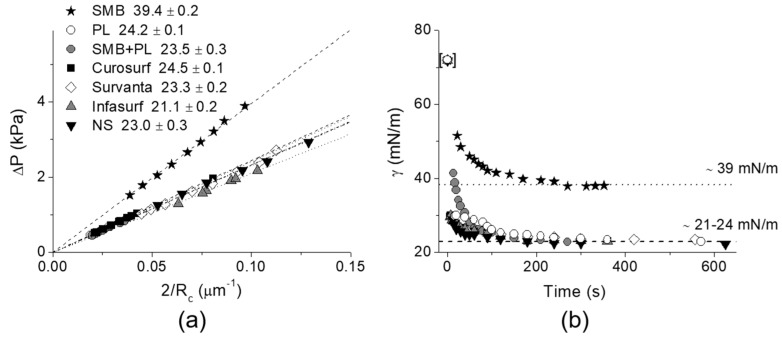
Equilibrium air-water surface tensions for lung surfactants in Tris buffer (pH 7) at 37 °C. The data include 1 mg/mL suspensions of three different commercialised lung surfactants and extracted native surfactant (NS) from porcine lungs and the measurement of a synthetic lung surfactant formulation: 4% Super Mini-B (SMB) lung surfactant protein peptide in a phospholipid mixture (PL = DPPC:POPC:POPG 50:30:20 molar ratio), and the pure SMB peptide itself. (**a**) Equilibrium surface tension plot from the applied pressure vs. 2/*R_c_* plot. The surface tension for each sample are shown in the legend derived as usual form linear fitting of the slopes. (**b**) Dynamic surface tension change of each sample by using the first-generation area expansion method developed by Lee et al. [9]. The symbols correspond between these two graphs. With permission from American Chemical Society [10].

Firstly then, Figure 24 shows both equilibrium and dynamic surface tension data of the various different types of lung surfactants at the air water interface in the tapered micropipette [10]. The equilibrium surface tension values obtained from the slope of a linear fit to the Δ*P* vs. 2/*R_c_* plots are shown numerically in the legend (Figure 20a). 

Interestingly, the animal-derived lung surfactants (protein-free Curosurf, Survanta, and protein-containing Infasurf [147]) and extracted native porcine lung surfactant (NS) all gave surface tension values in the range 21–25 mN/m. Importantly, theses surfactants showed the same equilibrium surface tension values as the pure phospholipid mixture (PL = DPPC:POPC:POPG 50:30:20 molar ratio), ~24 mN/m (see earlier Figure 18 and Figure 19). Consistent with this data, mixing the synthetic peptide SMB [10,179] in with the artificial lipid mixture (PL) did not change the equilibrium surface tension, which was 23.5 mN/m. In the absence of lipids, Figure 20a also shows that the peptide itself had a slightly higher surface tension of 39.4 mN/m. Thus, even though they each contain either some of the native proteins or the synthetic peptide for all compositions, the equilibrium surface tensions were all dominated by the lipid fraction. 

The question then was, is there an effect of the inclusion of protein or peptide on the dynamic surface tension? As shown in Figure 20b, the dynamic surface tension measurements (indicated by the time-rates of change of surface tension and so in units of mN/m/min) for each of the surfactants gave initial adsorption rates in the range of 60–240 mN/m/min, over the first 30 s. These values were again in the same range as that of the pure phospholipid mixture (PL), and in agreement with the single pure phospholipid solutions, maximum ~50 mN/m/min (Figure 19). Interestingly, as shown in Figure 20b, the SMB peptide itself also showed a similar dynamic adsorption speed, ~60 mN/m/min.

#### 6.3.3. Morphological Changes of Membranes Formed at the Interface

The most interesting and biologically-relevant aspect of the study was when we then explored the morphological changes that were observed during some of these equilibrium and dynamic measurements. We had seen not only adsorption but actual growth of structures from the lung surfactant-adsorbed surface. These studies, carried out for pure phospholipid and the various protein and peptide containing formulations, demonstrated quite categorically that, especially the SMB peptide was causing massive rearrangement of adsorbed lipid layers at the air-water surface.

**Figure 25:**
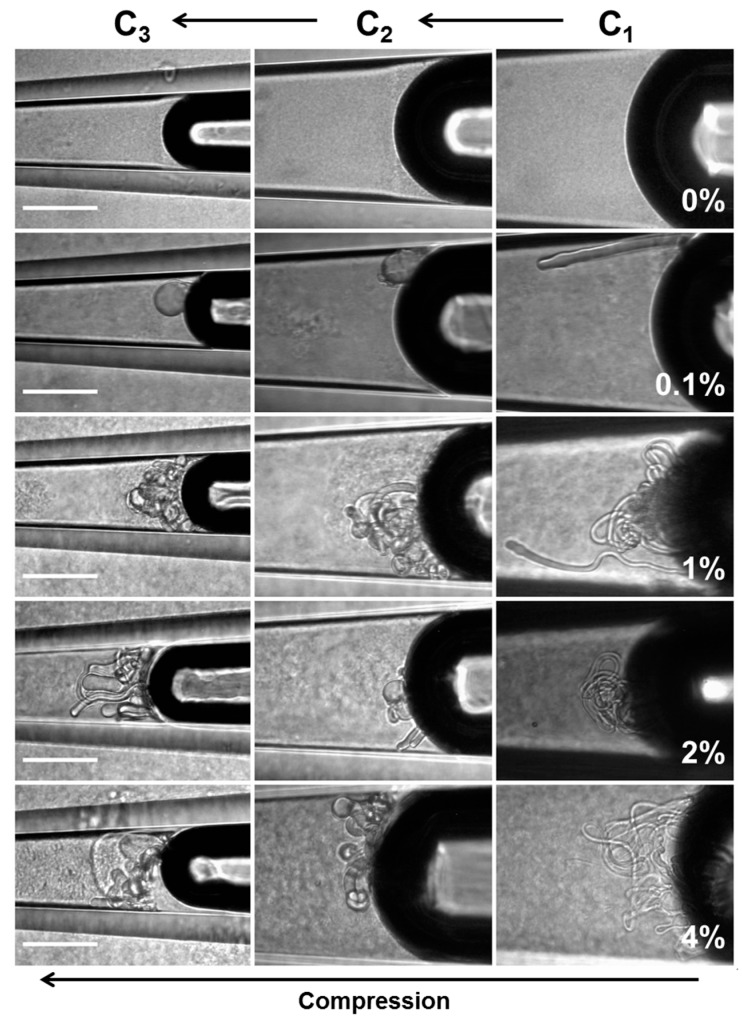
Microtubules formation and growth at 37 °C. Air-water surfaces are shown in the tapered micropipette for a series of increasing SMB concentrations from 0 to 4 wt% in the Phospholipid (PL) mixture solution. Each meniscus is moved down the micropipette taper by increasing the micropipette pressure from 0.7, 1 and 2 kPa, to give the sequential surface compressions C1, C2 and C3, respectively. In each case, compressions were made, and images were obtained after a 10 min waiting period. The scale bar is 50 μm. With permission from American Chemical Society [10].

As the interface was trapped inside the tapered pipette and could be viewed under high magnification, we observed dramatic and complex morphological changes of the monolayers and subsequent membranes formed at the interface for the SMB + PL mixture. Figure 25 shows the kind of microtubule-formation and growth at the interface of this totally synthetic SMB formulation that was triggered by compression of the interface. Under increasing compression (0.7, 1 and 2 kPa), microtubule-growth was only observed from SMB-containing lipid samples. The pure lipid (0 wt% SMB) maintained a clean monolayer surface. However then, with increasing SMB concentration above 0.1 wt% SMB in the mixture suspension (1, 2, and 4 wt%), the lung surfactant showed a stronger and stronger tendency for tube-formation-activity and dynamics. Although not as dramatic, similar microtubule-forming behaviour was observed for the protein-containing Infasurf and NS, but not for the protein-free liposomes Survanta and Curosurf [147,181].

Thus, the inclusion of protein in the commercial formulations, and especially the SMB peptide in Super Mini-B, was shown to be a key factor for the microtubule formation. While previous analysis had yielded some understanding [182], these new micropipette data gave a more detailed knowledge of how certain peptides (and proteins) interact (adsorb, intercalate, bond, and fuse) with lipids within lung surfactant multilayers. They provided a deeper physico-chemical understanding of mechanisms that drive their biological function.

Length and volumetric growth rates were calculated from the averaged slopes of plots of tube diameters, lengths, and volumes versus time of the microtubes growing from the surface lipid layers containing increasing SMB concentrations, from 0.1 to 4 wt%. Data from this experiment are presented in Table 3. Measurements were made of several tubular structures whose growth from the surface membrane layers was visualised in real time inside the micropipette and are listed as a function of SMB content in the aqueous phase suspension of DPPC:POPC:POPG (50:30:20) liposomes. As seen in Table 3, the length- and volume-growth rates were all positive and relatively consistent for each SMB concentration, at 2–3 μm/s in length and 20–30 μm^3^/s in volume. However, there were relatively large standard deviations on the same order as their average sizes. Tube-growth measurements were made over time-periods of 60–120 s, and then, interestingly, the tubes tended to retract back and aggregate as more spherical structures. Tube-volumes were estimated by multiplying the instantaneous length by the apparent cross-sectional area of the tube, calculated from the measured diameters at the equatorial plane of each structure. 

**Figure 26:**
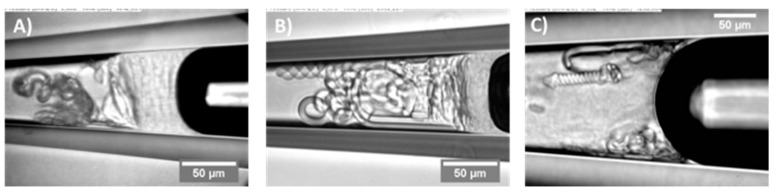
Video Micrographs showing presentative membrane structures observed in lipid-Minisurf samples. (**A**) Stacked and collapsed membrane multilayer layers beneath the interface; (**B**) protrusions and tubules growing from the surface layers, (**C**) independent helical structures resulting from the coiling of membrane microtubes. Higher levels of material accumulation next to the interface led to an increased number of tubes and helixes developing towards the water side (see Supporting Information and videos [183]).

One of the most astonishing observations in the micropipette experiments was how the adsorption of the SMB peptide-lipid system became a “living” multilamellar structure [10]. As shown in Figure 25, and in additional images in Figure 26, we saw inside the pipette that new tubular and then helical structures could grow out of the multilamellar stacks driven, we assume, by the peptide-lipid causing massive membrane membrane-adhesion, fusion and aggregation

Interestingly, the peptide is overall positively charged and the inclusion of the negatively charged lipid POPG brings the possibility of close electrostatic interaction, charge-neutralisation between membranes and the distinct potential of bilayer-bilayer fusion. Thus, these peptide-lipid mixtures when presented to an air-water surface were indeed able to illicit membrane-membrane fusion and the complete rearrangement of the multi-bilayers into such relatively dehydrated and complex membrane structures. The reader is encouraged to go to the online version of the paper [10] and view the available videos [184,185] in Supporting Information [183].

The presence and dynamic-growth of these new lipid-peptide multilamellar and tubular structures could well be foundational to their action in vivo at the lung-air surface. Our observations show that the tubules and multilayers grow into the aqueous space from the air-side of the surface and, so in vivo, they could perhaps integrate and embed themselves into the lung epithelium. Their active growth could actually be the basis for new innovation for drug delivery that could penetrate into the lung epithelium and so deliver any encapsulated drug deeper into this interfacial tissue.

### 6.4. Interfacial Tension is a Key Parameter in Nanoprecipitation

This next application of interfacial tension measurement inside the tapered pipette was motivated by the need to measure the interfacial tensions of Triolein (TO) against ethanol-water mixtures, including in the presence of phospholipid, palmitoyl-oleoyl-phosphatidylcholine (POPC, C16:0–18:1). In a series of separate projects, we have been studying and characterising the nanoprecipitation of Triolein from organic solvent into the anti-solvent water. This rapid solvent-shifting technique forms the basis for our new thrust in anti-cancer drug delivery to metastatic tumours [186]. Cancers have an altered lipid-metabolic-reprogramming [187], over-express Low Density Lipoprotein Receptors (LDLR) [188] and take in more LDLs and albumin than normal cells, to the extent that a cancer patient’s LDL- and albumin-counts can even go down [189]; LDL-uptake promotes aggressive phenotypes [190] resulting in proliferation and invasion in breast cancer [191], and an abundance of LDL-Receptors is a prognostic indicator of metastatic potential [192]. Inspired by the LDL and its biology-of-uptake by cancer cells, Needham’s lab has developed a strategy we call, “Make the drug look like the cancer’s food” [186] and are currently developing and testing new prodrug nanoparticles in cell and preclinical animal studies.

We have used Triolein and the POPC lipid as test materials with which to understand the fundamentals of such nanoprecipitation techniques. The key to understanding the process is to start with Classical Nucleation Theory (see excellent review by Karthika et al. [193] and applications to hydrophobic materials by Horn and Reiger [194]) and to measure the two main free parameters for a particular system. These are: the degree of supersaturation (*S*) of TO in the EtOH/Water mixture in which it becomes supersaturated and therefore precipitates out; and the interfacial tension (*γ*) of the TO at the same concentration. These two parameters, along with the molecular volume of TO (*V_m_*) (and $k_{b}$T) give a direct measure of the critical radius (*r_c_*) of the precipitated nucleus material, such that,

(10) $$r_{c}=\frac{2\gamma V_{m}}{k_{b}TlnS}\;.$$

In our drug delivery design, we coat the nanoparticle with a monolayer of lipid that stabilises the new nucleate and kinetically traps it at the minimum size. Interestingly, just like TO, the phospholipid is soluble in pure ethanol and in high % ethanol in the ethanol-water mixture. At some point in the solvent exchange (i.e., exchange of the solvent ethanol for anti-solvent water), it also comes out of solution to form the monolayer around the precipitated TO (or drug) nucleate. This micropipette experiment then was designed to measure the interfacial tension of a series of EtOH/Water mixtures without and with the presence of the phospholipid POPC; the challenge was to measure the interfacial tension in the tapered micropipette for this liquid-liquid system where one of the components (ethanol) is volatile.

#### 6.4.1. Tapered Micropipette Manipulation Technique for Volatile Oil-Water System (Water-Ethanol Mixtures)

**Figure 27:**
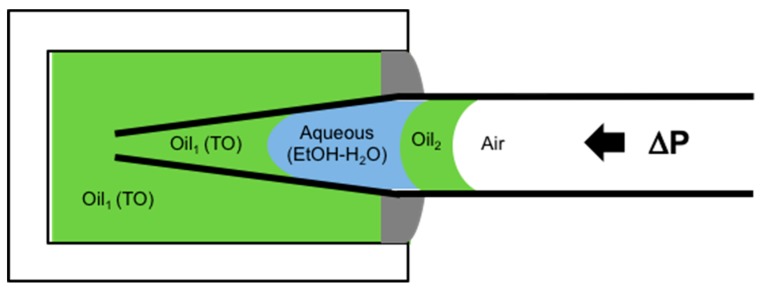
Schematic illustration of the equilibrium interfacial tension measurement of an oil-water interface in which one component is volatile [16,17]. In the set-up, the chamber is filled with the oil solution (Oil_1_) Triolein. Then, the tapered micropipette, is prefilled with the same oil (Oil_2_) and the water solution under investigation is also aspirated into the tip. The pipette is then inserted into the Oil_1_ under positive applied pipette-pressure in order to maintain the water and Oil_2_ plug inside the tapered micropipette. The Oil_2_ solution is used to avoid evaporation of water solution. Once again, control of applied pipette pressure drives the interface meniscus to measurable radii in the tapered pipette, and the application of the Laplace equation, Equation (5), gives the interfacial tensions.

In this experiment, we managed to maintain the starting ethanol concentrations in the ethanol water mixtures by producing liquid “plugs” that sealed the phase interfaces being measured inside the tapered pipette [16,17]. Figure 27 shows a schematic of the liquid-liquid equilibrium interfacial tension measurement system for Triolein measured against water-ethanol mixtures that also contains phospholipid. Thus, following the procedure in the figure legend, the aqueous solution is “trapped” between two oil solutions, Oil_1_ in the chamber and Oil_2_, by using a plug that is preloaded into the pipette to prevent evaporation of the ethanol component during the measurement. As illustrated, such a set-up contains at least three different interfaces, i.e., Oil_1_-water, water-Oil_2_, and Oil_2_-air that each can influence the way the applied pressure is dropped across the system, depending on their interfacial tensions and their respective radii. Therefore, dealing with this kind of system is not trivial (experimentally as well as theoretically).

As reported in Utoft’s thesis [16], and described in detail in [17], to obtain the interfacial tension of this particular Oil_1_-water interface, we needed to know the pressure difference for each of the three interfaces: Oil_1_-Water, (*O*1,*W*); Water-Oil_2_; (W,O2); and at the back of the micropipette, Oil_2_-Air (*O*2,*A*), and where, the corresponding pressure differences are $\Delta P_{O1,W}$, $\Delta P_{W,O2}$, $\Delta P_{O2,A}$

It is instructive to go through this analysis for any application where multiple interfaces and components are included, such as may occur in a microfluidic system. 

The pressure differences at each interface then are:

(11) $${\mathrm{Oil}}_{1}-\mathrm{Water}:\;\Delta P_{O1,W}=P_{W}-P_{O1},\;\mathrm{Water}-{\mathrm{Oil}}_{2}:\;\Delta P_{W,O2}=P_{O2}-P_{W},\;{\mathrm{Oil}}_{2}-\mathrm{Air}:\Delta P_{O2,A}=P_{A}-P_{O2}.$$

Therefore, starting at the back of the pipette, the applied pressure going from the air inside of micropipette all the way to Oil_1_ in the chamber is the sum of these pressure differences as follows,

(12) $$\Delta P=\Delta P_{O2,A}+\Delta P_{W,O2}+\Delta P_{O1,W}=P_{A}-P_{O1}=\Delta P_{O1,A}.$$

Following the Laplace equation for the tapered micropipette, Equation (5), the surface tension between water and Oil_1_, *γ_O_*_1,*W*_, is thus expressed as,

(13) $$\Delta P_{O1,W}=\Delta P-\left(\Delta P_{W,O2}+\Delta P_{O2,A}\right)=\frac{2\gamma_{O1,W}}{R_{O1,W}}.$$

Therefore, to obtain the *γ_O_*_1*,W*_ value for a single surface tension measurement, requires measuring the interfacial pressure of Δ*P_W,O_*_2_ and Δ*P_O_*_2*,A*_. However, if the sum value, Δ*P_W,O_*_2_ + Δ*P*_O2,A_, of each pressure is constant, the surface tension of γ_O1,W_ can be calculated from the slope of Δ*P* vs. 2*/R_O_*_1*,W*_ plot (see Figure 7c and Figure 9). The intercept at the Y-axis (at 2/*R_O_*_1*,W*_ = 0) is equal to the constant value of Δ*P_W,O_*_2_ + *ΔP*_O2,A_. Thus, if the intercept is zero, it means that Δ*P_W,O_*_2_ + Δ*P*_O2,A_ become zero, as in the liquid-gas interfacial tension result in Figure 7c. Therefore, to keep the value of Δ*P_W,O_*_2_ + *ΔP*_O2,A_ constant during the measurement, the two interfaces of water-Oil_2_, and Oil_2_-air must be kept in the non-tapered (large-diameter-bore, 450 μm) micropipette section during the measurement. Hence, the non-tapered part is quite large compared to the tapered pipette where the measurements are made, making the pressure drop at this diameter comparatively low to maintain an interface in this region of the pipette. Therefore, both interfaces are kept in the constant inner diameter section by adjusting the volume of water solution in the pipette and applying the appropriate pressure. As is quite obvious then, in order to obtain the *γ_O_*_1*,W*_ value, the interface under measurement, of Oil_1_-water, has to always be in the tapered section of the micropipette tip.

#### 6.4.2. Lipid Adsorption at the Triolein-Ethanol/Water Interface

**Figure 28:**
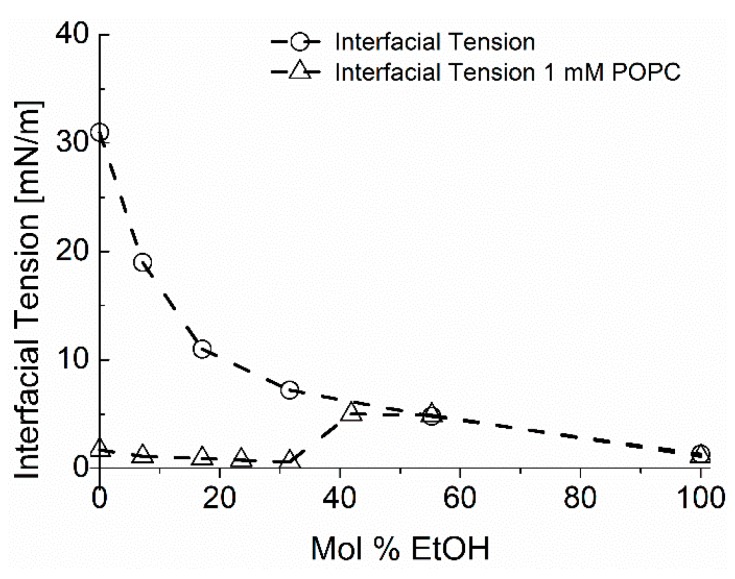
Interfacial tension of Triolein vs. ethanol-water mixtures across the entire binary regime in absence and presence of 1 mM palmitoyl-oleoyl-phosphatidylcholine (POPC). Open circles show the interfacial tension of Triolein against water-ethanol with increasing ethanol mol%. Open triangles show the measured interfacial tension of Triolein against water-ethanol in the presence of 1 mM POPC, present as liposomes in the aqueous solution. The sharp bend for the interfacial tension in the presence of POPC at around 40 mol% ethanol represents the limit of solubility of POPC in the ethanol-water mixture.

This new advanced tapered micropipette manipulation technique was then used to measure the Triolein-water interfacial tension and to investigate palmitoyl-oleoyl-phosphatidylcholine (POPC, C16:0–18:1) monolayer formation at the same Triolein-water interface. Triolein is an unsaturated triglyceride, having three oleic acid (C18:1) acyl chains and so forms an immiscible interface with water with perhaps an oriented monolayer of Triolein at this interface, but itself it is not that surface active and so can easily be replaced by adsorbing phospholipid. Figure 28 shows the interfacial tension between Triolein and ethanol-water mixtures in the absence and presence of 1 mM POPC. 

For pure Triolein against increasing ethanol concentration in the ethanol-water mixture, the interfacial tension of the oil-water interface showed a smooth decrease from *γ* = 31.1 ± 0.1 mN/m for the pure water to 1.3 ± 0.1 mN/m for pure ethanol. Hence, Triolein forms an immiscible phase boundary with a finite surface tension against water, and against ethanol. As mentioned in Section 3, short-chain alcohols can themselves decrease the interfacial tension by forming a monolayer that then reduces the Triolein interfacial tension.

When POPC was mixed in the water, the interfacial tension was dramatically reduced from 31 mN/m for the pure Triolein-water interface to 1.7 mN/m for the formed POPC monolayer. Thus, as we have seen before for lipids at air-water and now oil-water interfaces, POPC is a very effective way to reduce the oil-water interfacial tension by forming a POPC monolayer. This value is similar to that measured by Needham and Haydon for GMO at the water-Triolein interface of 1.82 mN/m [1]. With increasing ethanol concentration in the water-ethanol mixture the interfacial tension was even further decreased to just 0.6 mN/m, showing that there was some influence of adsorbed ethanol along with the phospholipid. Then, at a critical ethanol concentration of between 32–42 mol%, the interfacial tension actually rose to a value of 5.0 ± 0.1 mN/m, coincident with the lipid-free interface. Above 55 mol% ethanol, the interfacial tension followed the exact same trend as in the system without POPC present. 

What this interfacial tension behaviour therefore seems to indicate is a limiting solubility of POPC in ethanol water, i.e., at ethanol concentrations below 42 mol% the POPC is still insoluble in this mainly aqueous mixture, but above 42 to 45 mol% ethanol, the liposomes are solubilised in the monomer state [195]. Once dissolved, the POPC does not account for any further adsorption to the interface. Rather, ethanol, having a much higher surface activity than the dissolved lipids [196], replaces POPC molecules at the Triolein surface and take over the role of the main surface-active compound by this exchange. Thus, in any microfluidic or other interfacial systems, it is necessary to quantify these tensions as a function of solvent mixtures and determine which of the potentially surface active components is actually adsorbed and determining the surface or interfacial tension. Once again, this is where the micropipette technique can isolate individual components and make these crucial measurements.

### 6.5. Microsurface Tensions and Droplet Dissolution for Microfluidics 

Finally, not only can we use the pipettes as a tapered tube for surface and interfacial tension measurements, the micropipette manipulation technique is capable of forming and making fundamental measurements and analyses for single (individual) particles. Such an example was given previously for the water-in-oil experiments of Yeung, but we have many studies and papers on this aspect of the pipette technique including droplet dissolution of one immiscible phase into another, as well as precipitation and crystallisation from such concentrating solutions [12,95,164,165,168,197,198,199]. These kinds of experiments are critical for establishing processing parameters in systems such as microfluidics and homogenisation. In order to demonstrate what can be achieved at the single particle level in the case of microfluidic processing, we present here a brief description of some experiments we carried out and reported in a paper directed at this same audience, entitled, “From Single Microparticles to Microfluidic Emulsification: Fundamental Properties (Solubility, Density, Phase Separation) from Micropipette Manipulation of Solvent, Drug and Polymer Microspheres” [7], as well as in other studies and reports that form a series of industrial collaborations (not published). It is here that interfacial tensions can change as a result of adsorption of surfactants and polymers, or drugs, and these materials can themselves dissolve into the second (usually) aqueous phase. These experiments also give a measure of the diffusion coefficients of each component, and provide a correlative measure of diffusion that has agreed with dynamic surface tension measurements we have also made with the micropipette technique, as described earlier in Section 4.4.1. for octanol.

One good example where interfacial tensions, droplet dissolution, and drug and polymer precipitation are combined is microfluidic processing of polymer microspheres for drug encapsulation. In these systems one of the most used and studied materials is that of a biodegradable PLGA polymer particle, where, in processing, drug is co dissolved in the PLGA-solvent (usually Dichloromethane) solution. What we have provided with the micropipette technique [18], is:

- An ability to measure all relevant interfacial tensions for each component that tend to be unique to the industrial process and so are not readily obtained from the literature,
- Make individual particles as a function of composition,
- Observe any emerging microstructures inside or on the particles,
- Make measurements of fundamental properties like mechanical deformation of these materials at the single microparticle level and,
- Measure any dissolution (e.g., diffusion coefficients) into the suspending medium.

**Figure 29:**
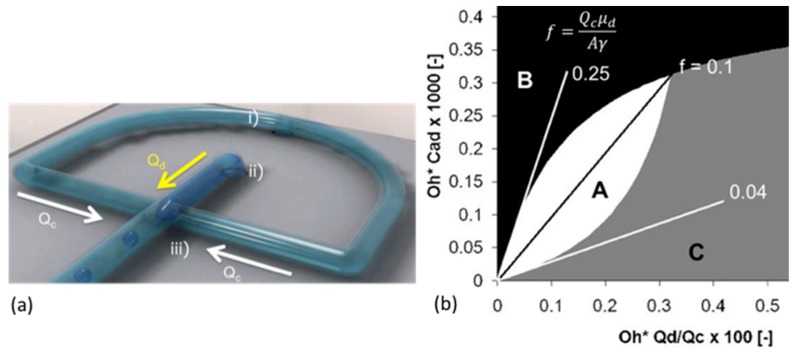
The microfluidic system and flow parameters that determine effective droplet formation. (**a**) Schematic 3D picture of droplet formation showing the arrangement of the two inlet tubes: (i) continuous phase inlet that then splits into two tubes; (ii) dispersed phase inlet; (iii) mixing head. (bottom) High-speed microscope image of the single-channel production window. (**b**) Flow map, depicting all combinations of liquid properties, microchannel size and flow rates. Combinations in the white area (region A) yield the desired process, i.e., spherical particles; combinations in the black area (region B) lead to instabilities and non-uniform droplets; and, in the grey area (region C), to droplets larger than the exit channel. From Kinoshita et al. [7].

As discussed by de Bruijn et al. [200], an accurate measurement of interfacial tension is important for setting the right parameters for microfluidic droplet formation. The process of microfluidic emulsification involves the injection of two solutions: the dispersed solvent phase (the liquid to form the droplet containing the polymer and any API, flow rate Qd); and the aqueous continuous phase (the carrier liquid surrounding the droplet, also called the dissolution medium, flow rate Qc). As shown in Figure 29, the dispersed phase is injected through the central inlet, indicated as (ii), which is continuous with a cross-junction geometry that connects it to the outer cross-inlet, labelled as (i), through which the continuous phase flows. 

The dispersed phase is pumped into a micro-channel and enters the cross junction, ‘hydrodynamic flow focusing’ of the continuous phase breaks the polymer solution into microdroplets. Thus, parameters such as viscosity, *μ*, flow rate, *Q*, and interfacial tension, *γ*, become important in how and at what rate droplets form. The key is a precise control over the flow rate Q_c_ and *Q_d_* from the inlet channels (Figure 29a), and control of the process lies in the force-balances at the intersection: the balance between interfacial and viscous forces (characterised by the capillary number of dispersion phase, *C_ad_*); and the balance between the dispersed phase and the continuous phase that is forced into the same outlet (characterised by the ratio of flow rates *Q_d_*/*Q_c_*). The dimensionless parameter of *Q_d_*/*Q_c_* is often cited to show the profile of characterised microdroplet distribution such as the flow map (Figure 29b). In region A, the produced microparticles become nicely spherical. However, in region B the microdroplets become non-uniform and in C can be larger than the exit channel.

It is here where a knowledge of the interfacial tension γ is required for the two solutions used in the device. For the process of creating an emulsion it is preferred that the ratio of the dispersed phase flow rate *Q_d_* to the continuous phase flow rate *Q_c_* is ≤0.00272 Oh*, where Oh* is the Ohnesorge number of the system, and is inversely proportional to the interfacial tension *γ*.

The interfacial tension is clearly expected to depend on the nature and the concentrations of each component and so needs to be measured at their microscale interfaces for each solvent-polymer-drug-aqueous solution systems including with and without the stabilising surfactant polyvinyl pyrrolidine (PVA). For example, for control systems of pure solvents against the aqueous phase, such as DCM vs. water, the interfacial tension was measured to be 31.4 mN/m. The inclusion of 1 mM SDS in the aqueous phase to help with droplet colloid-stability, as expected, lowered this interfacial tension to 16.9 mN/m. For example, the inclusion of a particular drug (proprietary Pharma compound) at 25 mg/mL in the DCM vs. 1 mM SDS showed an average surface tension, of 13.4 mN/m. The interfacial tension of a 20 mg/mL PLGA and 2 mg/mL of an AI compound mixed in the final solvent mixture of DCM:DMSO (8:2) against water was 7.0 ± 0.2 mM/m but 10.5 ± 0.2 mN/m with 0.1% PVA added. These are the interfacial measurements then that allow parameters to be set to achieve effective microdroplet formation in the microfluidic device.

As mentioned, a knowledge of the dissolution time of microdroplets is also important in the latter stages of microsphere formation. Our micropipette techniques have also therefore been used to determine this time, as well as measures of the diffusion coefficients of solvents and also drugs. For example, the diffusion coefficient of DCM in the aqueous phase was measured by droplet dissolution and applying the EP Equation (8), to be *D* = 1.77 ± 0.01 × 10^−5^ cm^−2^/s, while the diffusion coefficient of the drug Ibp in PBS (pH 7.4) at room temperature, was three times smaller at *D* = 5.5 ± 0.2 × 10^−6^ cm^2^/s. That is, for a DCM-Ibp solution microdroplet, once the DCM had all dissolved, the pure drug continued to dissolve but at a much slower rate. Additionally, the time, *tD*, for a given diameter of a spherical microparticle of Ibp to dissolve in an infinite medium is given by the following equation derived from the EP equation [12,18],

(14) $$t_{D}=\frac{R_{o}^{2}}{2D}\;\frac{\rho }{C_{s}},$$

where, *R_o_* is the initial radius of the particle, ρ is the density of the Ibp (1030 mg/mL), *D* is the diffusion coefficient of the molecule in aqueous media (5.5 ± 0.2 × 10^−6^ cm^2^/s), and *C_s_* is its solubility limit in PBS (pH7.4) buffer, 0.825 mg/mL (4 mM). This equation successfully predicted the measured dissolution time of a pure Ibp microparticle of diameter of 16.4 μm, as ~75 s and dissolution rate of 30 femtogram/s or 1.9 picograms/min [18]. This calculation shows how important it is in applications of pure drug dissolution to not only know the size of the drug microparticles but to actually control it. Using the micropipette technique, the dissolution rates for pure drug microparticles can be measured in water, buffer, surfactant solution or biological fluids (e.g., synovial fluid or blood plasma).

Thus, by simply using the dissolution time equation derived from the EP model, we can provide our microfluidic collaborators with a measure of the time needed in the microfluidic processes for solvent, drug, or polymer dissolution and how this changes as a function of any processing parameter. These may include any composition change in the organic-polymer-drug solution or the addition of stabilising surfactants in the aqueous phase. It can be done for a range of microparticle diameters from 5 to over 100 μm. In one application, we showed that the dissolution time of a very hydrophobic drug meant that the formulation did not in fact need a polymer-releasing system. EP and *t_D_* calculations simply showed that for a microsphere diameter size of 2 μm, it would take only 2.1 min for complete dissolution. However, for a 600 μm diameter microsphere (300 times larger diameter) it would take a much longer time, on the order of 6 months. The required specifications of the formulation was a release (or now dissolution) time of ~6 months, and so this was achieved with just drug microparticles. Such a formulation was superior to any PLGA encapsulation where the PLGA itself would be degraded in few weeks to a month.

## 7. Summary and Conclusions

In this review paper then, we have presented a series of the micropipette techniques that we have developed and used to make measurements of the surface and interfacial tensions for clean air-water surfaces and oil-water interfaces. By using a tapered micropipette and introducing rapid image monitoring of the position of meniscus in the micropipette and hence its radius of curvature for a given applied micropipette pressure, we have presented a series of equilibrium tensions as well as equilibrium and dynamic tensions due to the adsorption of water-soluble surfactants and water-insoluble and lipids. Micropipette dimensions associated with the capillary tip are ~5–10 μm, and the micropipette can taper out to 450 μm, thus, importantly all measurements are actually made at the microscale. Following the Young–Laplace equation and geometry of the capillary, the surface or interfacial tension value is simply obtained from the radius of the meniscus in the tapered pipette and the applied pressure to keep it there. 

As an interesting prelude to the comprehensive description of these experimental techniques and their theoretical analysis we also provided a brief potted-historical perspective that included Franklin’s early experiments that demonstrated molecularity and monolayer formation, fundamental surfactancy driven by margarine, the first use of a micropipette to (circuitously) measure bilayer membrane tensions and free energies of formation, and how this black lipid film concept formed basis for study and applications of membrane ion-channels in Droplet Interface Bilayers and beyond, into printed microdroplet “tissues.” 

To address the goals of the special issue we selected five examples of where our measurements have had an impact on applications in micro-surfaces and microfluidics, including gas microbubbles for ultrasound contrast; interfacial tensions for micro-oil droplets in oil recovery; surface tensions and tensions-in-the surface for natural and synthetic lung surfactants; interfacial tension in nanoprecipitation; and micro-surface tensions in microfluidics. The micropipette technique that was originally designed and built to study the properties of biological cells (red and white blood cells and Giant Unilamellar-Lipid Vesicles) by Evans, Kwok and Needham, and Chien, Skalak, Schmidt-Shoenbein and co-workers, has now found new and myriad utility in making as well as characterising individual and pairs of micro colloids and surfaces, and is an essential tool for anyone studying, designing or using Micromachines for science and technology of small structures, devices and systems. We have tried to show how important and versatile this micropipette technology can be especially as soft matter is miniaturised, surface areas become huge, and their surface energies become major drivers of structural and material transformations in micro- and nano-systems. As we have also tried to show, equilibrium surface and interfacial tension data has also to be coupled to dynamic processes, such as adsorption of simple surfactants or more complex insoluble materials like lipids (and this would also include future studies on surface and interfacial polymers and proteins), and the dissolution by diffusion (or convection) of the tiny microscale materials (often in the volumes of pico- and femto-litres), characterised by diffusion in unique and perhaps not-already-known solvent systems.

Even though it is a very long document, we think that the whole structure and flow brings a readable and educational/informative review of these important techniques. It takes us from a historical perspective that includes a personalised approach to some aspects of surfactancy, through the basic techniques we have developed and a series of five applications that we hope are of interest to the Micromachines readership (and beyond). We therefore offer this unique capillary- and single-particle-manipulating techniques to the Micromachines, and Microsurfaces community to help enhance the development, design, and testing of various applications such as the stated: “microfluidic and lab-on-chip devices, soft gripping and manipulation of particles, colloidal and interfacial assemblies, fluidic/droplet mechatronics,” and the fundamentals or applications yet to be explored. 

## Figures and Tables

**Table 1:** Alkane and alkanol interfacial and surface tensions against water or air. Micropipette aspiration technique results are shown in bold letters. Data are at 20 °C, except, micropipette aspiration technique results (23 °C), γ_OW_ of Decane (24.5 °C), and both 1-Hexanol interfacial tensions (25 °C). The literature data is obtained from [91,92].

**Alkane**	**Alkane-Water** **γ_OW_ (mN/m)**	**Alkane-Air** **γ_OA_ (mN/m)**
Hexane (C6)	51.1	18.4
Octane (C8)	50.8	21.6
Decane (C10)	**51.2** (52.0)	**23.6** (23.8)
**Alkanol**	**Alkanol-Water** **γ_OW_ (mN/m)**	**Alkanol-Air** **γ_OA_ (mN/m)**
1-Hexanol (C6)	6.8	25.8
1-Octanol (C8)	8.52	27.5
1-decanol (C10)	8.97	28.9

**Table 2:** Dynamic surface tension measurement techniques and their possible time-ranges for surfactant adsorption and area characteristics (adapted from Eastoe [135] and now including our new Micropipette Interfacial Area-expansion Method (MIAM) technique).

Techniques	Short-Time Adsorption (s)	Interfacial Area (µm^2^)	Functions and Drawbacks	Ref.
MIAM	<1	10^3^–10^5^	Interfacial area expansion 150–200 times No requirement of contact angle information Leakage for high viscous and sticky materials for glass surface	[6,9,7,8]
PBT	10^−1^	10^7^	Reliable data in the middle time range (0.1 s to mins) Limit of long-time adsorption (bubble detachment) Bubble expansion rate increasing cause error	[139,140]
Growing-drop	10^−2^	10^7^	Wide range (milliseconds to more than hours) dynamic surface tension measurement Need to keep constant flow, material loss from tip climbing	[141,142]
Oscillating Jet	10^−3^–10^−2^	10^5^	Short time adsorption measurement Not suitable for the long-time adsorption range of (>10 s), no equilibrium tension	[87,143,144]
MBPM	<10^−3^	10^5^	Wide range, short-time and long-time, adsorption measurement Leakage of material, requirement of hydrophobic treatment inner wall and hydrophilic tip	[133,136,145]
Langmuir–Wilhelmy	>20	~10^2^ cm^2^–m^2^	Easily assembles with microscopy and spectroscopy methods Not suitable for fast area exchange because of leakage of material Contact angle requirement	[146,147]
CBS	~10^−1^	10^7^	Leakage proof, mimic alveolar environment Limited interfacial area expansion (two times) range for adsorption dynamic	[148,149]
PBS	1	10^6^	Mimic breath control, easy to operate Material leakage, lacks operational flexibility	[122,150]

MIAM: Micropipette Interfacial Area-expansion Method; PBT: Pendant Bubble Tensiometer; MBPM: Maximum Bubble Pressure Method; CBS: Captive Bubble Surfactometer; PBS: Pulsating Bubble Surfactometer.

**Table 3:** Diameters and linear and volumetric growth rates of the tubes in SMB + PL Samples. All the results are shown as the mean ± SD, calculated from all the tubes analysed for each composition. From [10].

% SMB	# Analysed Tubes	Thickness (μm)	Linear Growth Rate (μm/s)	Volume Growth Rate (μm^3^/s)
0.1	5	4.46 ± 1.85	1.76 ± 1.24	28.37 ± 28.37
1	15	3.41 ± 1.07	2.68 ± 3.84	22.38 ± 25.11
2	20	4.73 ± 2.19	2.23 ± 2.34	26.65 ± 14.10
4	21	3.48 ± 1.24	2.73 ± 1.70	22.14 ± 12.95

## Dedication

This article is dedicated to Needham’s two most influential mentors, D. A. Haydon FRS and E. A. Evans. Evans was also mentor to Kinoshita. We owe them so much. Thank you.
